# A deep learning-based fusion framework for robust fine-grained classification of sea turtles in support of marine biodiversity

**DOI:** 10.1371/journal.pone.0344942

**Published:** 2026-06-09

**Authors:** Parkpoom Chaisiriprasert, Apicha Deearom

**Affiliations:** College of Digital Innovation Technology, Rangsit University, Pathum Thani, Thailand; Sultan Qaboos University, OMAN

## Abstract

Accurate classification of sea turtle species is crucial for ecological monitoring and conservation, yet traditional visual classification methods remain limited by underwater imaging challenges such as occlusions, poor lighting, and background noise. To address these limitations, we propose an enhanced deep learning-based classification framework that integrates both color and structural features to improve the robustness of species recognition in complex marine environments. Building upon the ResNet-50 backbone, we introduce a four-channel input tensor comprising RGB data and Sobel-filtered edge maps, capturing both semantic and morphological information. Two novel fusion modules, LiteAFNet and AlphaBlendNet, are designed to integrate these features effectively. LiteAFNet leverages a lightweight attention mechanism to highlight discriminative regions, while AlphaBlendNet adaptively balances RGB and edge cues based on spatial context. Experimental results demonstrate significant improvements in classification performance across all evaluation metrics. Specifically, AlphaBlendNet achieves the highest precision (0.84), recall (0.88), F1-score (0.86), and mean average precision (mAP) of 87.2%, outperforming both the baseline fusion and LiteAFNet configurations. These results indicate that integrating color histograms with structural edge features enhances the model’s ability to distinguish between species with similar visual traits. This framework offers a scalable, accurate, and automated solution for underwater species classification and holds potential for broader application in marine biodiversity monitoring.

## 1. Introduction

Marine biodiversity plays a vital role in maintaining ecological balance, with sea turtles being among the most iconic and ecologically significant marine species. Accurate identification of sea turtle species is essential for population monitoring, conservation strategies, and environmental research. Traditional classification approaches rely heavily on expert visual inspection, which is time-consuming, labor-intensive, and prone to human error particularly in underwater environments where lighting conditions, occlusions, and complex backgrounds hinder visual clarity. As sea turtle populations face increasing threats from habitat degradation, pollution, and climate change, there is a growing need for automated, scalable classification systems that can assist in large-scale ecological monitoring.

Convolutional neural networks (CNNs), especially architectures such as ResNet-50, have demonstrated strong performance in image classification. These models learn deep hierarchical features, allowing them to recognize complex visual patterns. However, when trained solely on RGB images, they often struggle with fine-grained classification tasks such as differentiating sea turtle species with similar shapes and shell textures particularly in underwater conditions where color distortion and loss of detail are common. These limitations highlight the need to enhance input representations to capture more discriminative features. To address this, a classification framework is proposed that integrates two complementary visual cues: color and edge structure. Color histograms provide a global representation of chromatic distribution, aiding in distinguishing species with unique pigmentation. In parallel, Sobel filtering extracts edge maps that highlight morphological contours and fine structural details. These features are combined into a four-channel tensor consisting of RGB and edge information, enriching the model’s input with both semantic and structural content. Two fusion modules, LiteAFNet and AlphaBlendNet, are introduced to integrate these cues effectively. LiteAFNet uses a lightweight attention mechanism to emphasize key regions, while AlphaBlendNet adaptively blends RGB and edge features based on spatial context. Both modules are embedded into the ResNet-50 backbone, enhancing its ability to process enriched input and improving robustness in underwater image classification. This framework offers a practical direction for automatic sea turtle species recognition under real-world marine imaging conditions.

To contextualize the proposed framework, it is essential to examine existing work in marine species classification, image preprocessing, and multimodal fusion. Recent literature has explored a variety of deep learning architectures tailored to underwater visual challenges, as well as preprocessing pipelines designed to enhance low-quality marine imagery. Moreover, the integration of attention-based fusion techniques has emerged as a critical factor in improving model robustness and interpretability under complex aquatic environments. The following sections provide a structured review of these contributions, categorized into three domains: species classification, image enhancement, and fusion architecture.

### 1.1. Species classification

Foundational CNN-based pipelines established baselines for ecological image recognition and general visual classification [1,2]. Subsequent work automated cetacean photo-ID via contour extraction and species-distinctive cues including Risso’s dolphins and introduced standardized protocols for striped dolphins that facilitate automated workflows [3–5]. Deep detectors have been applied to sharks [6], and cross-domain evidence (e.g., mosquito larvae) highlights portability of image-based bio-ID workflows [7]. A systematic review consolidates machine learning and imaging methods for cetacean photo-ID [8], while modern detectors (YOLO-NAS/YOLOv8) show consistent performance for sea-lion detection under diverse field conditions [9]. Short reviews on fish classification outline architecture choices and data requirements [10].

For sea turtles, works span individual ID from postorbital keypoints [11], drone-based detection [12], and temporal learning for ecological data streams [13]. Recent applications include coastal green-turtle counting and tourism-facing conservation aids using deep learning [14,15]. Fine-grained/small-object scenarios leverage super-resolution with improved YOLOv8 for hermit crabs [16] and fused features with LIME-based interpretability for plankton [17]; related pipelines target marine microplastic characterization [18]. Robustness in underwater videos is improved via enhanced CNNs and motion-aware approaches for fish detection [19,20], while transfer learning and modern detectors enable multi-class recognition on limited datasets [21,22]. Species distribution modeling (SDM) adds environmental context for cetaceans and turtles [23]. At scale, improved feature alignment, template matching, UAV-oriented feature extraction, and database-level AI matching support re-identification in field data [24–27].

### 1.2. Image enhancement and preprocessing techniques

Underwater imagery typically suffers from color cast, haze/turbidity, low contrast, and noise, making preprocessing critical. Relevant components include feature-recognition algorithms and efficient edge extraction (e.g., parallel Canny/Sobel) to stabilize contours prior to learning [28,29]. Color-balanced histogram equalization and denoising-aware Sobel formulations sharpen boundaries while preserving textures [30,31]. Contemporary enhancement leverages multi-task fusion strategies [32] and synthesizes color-correction practices via systematic review [33]. Retinex-based networks improve visibility in low-light/turbid scenes [34]. Insights from adjacent domains (e.g., crack detection) inform preprocessing blocks that preserve fine textures essential for marine cues [35].

### 1.3. Fusion architectures and attention mechanisms

Attention-centric fusion improves cross-modal integration and interpretability. Transformer-style global context complements CNN attention modules such as CBAM, SE, and ECA, enabling adaptive saliency weighting and channel efficiency [36–40]. Lightweight/high-resolution backbones (Lite-HRNet) and split-attention networks (ResNeSt) balance accuracy with compute constraints [39,41]. Relational and cross-modal attention in person re-ID and RGB-D saliency demonstrates transferable patterns for marine settings [42,43].

Residual/semantic-aware designs (RFN-Nest, THFuse) and unified/unsupervised frameworks (U2Fusion) show that hybrid global–local mixing preserves structure under adverse conditions [44,45,46]. Underwater-specific pipelines (ACCDF) and “fusion-in-the-loop” strategies tailor fusion to downstream tasks [47,48]. Global–local networks for underwater enhancement (GL-Net with compressed-histogram equalization) illustrate benefits of hierarchical attention and contrast normalization in submerged scenes [49]. For fine-grained classification, pairwise/self-refining attention, dynamic convolution, and attention-pyramid pooling strengthen discriminability on subtle cues [50–52]; multi-scale/multi-attention designs in complex scenes offer lessons for cluttered marine environments [53]. Dual-attention fusion (SDAM) and cross-modal global-awareness designs (RGB–Thermal) address domain shift and heterogeneous sensing, supporting robust and scalable field deployment [54,55].

Evidence gap. End-to-end evaluations that jointly optimize enhancement–fusion–classification under heterogeneous sensing (UAV/subsurface) and variable underwater quality remain scarce, limiting cross-context transferability and field robustness.

## 2. Materials and methods

### 2.1. Data collection

A dedicated image dataset of sea turtles was assembled to support the development of an automated fine-grained species classification model for marine biodiversity monitoring. Images were collected from publicly available repositories, including Roboflow and seaturtle.org. Each image is accompanied by an annotation file specifying the species label and bounding box coordinates for the annotated turtle, which were used for downstream processing. No pre-computed feature vectors were used; feature representations were learned directly from the raw images, and no feature dimensionality reduction (e.g., PCA) was performed. The final dataset comprises high-resolution images of seven sea turtle species: Green Turtle (Chelonia mydas), Hawksbill Turtle (Eretmochelys imbricata), Leatherback Turtle (Dermochelys coriacea), Flatback Turtle (Natator depressus), Loggerhead Turtle (Caretta caretta), Olive Ridley Turtle (Lepidochelys olivacea), and Kemp’s Ridley Turtle (Lepidochelys kempii).

**Fig 1:**
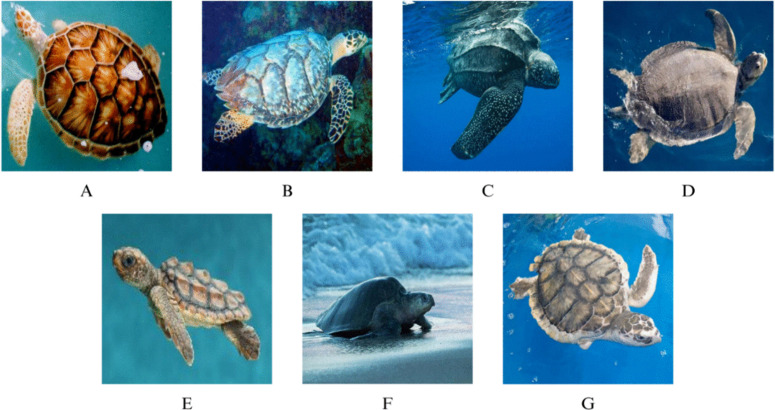
Examples of sea turtle species from the dataset. Images A to G correspond to: **(A)** Green, **(B)** Hawksbill, **(C)** Leatherback, **(D)** Flatback, **(E)** Loggerhead, **(F)** Olive Ridley, and **(G)** Kemp’s Ridley.

The selection criteria focused on the presence of identifiable carapace patterns, as the shell morphology provides essential visual cues for differentiating between species. The dataset emphasizes dorsal (top-down) views of the carapace but also includes lateral perspectives to capture variations in shell curvature and edge profile. A particular effort was made to include images taken under diverse real-world conditions, including underwater environments with different lighting intensities, shadow effects, and background complexity. These variations are critical in simulating realistic field scenarios where visibility may be affected by water clarity, light refraction, or camera angle. Fig 1 illustrates representative examples from each class, showcasing the diversity in shell patterns and imaging conditions. This visual reference serves to emphasize both inter-class variation and the challenges posed by underwater image variability.

To improve the dataset’s variability and robustness, a data augmentation process was applied. Standard image augmentation techniques were employed, including random rotation, scaling, and horizontal flipping. These transformations helped to synthetically increase the number of samples and expose the classification model to a broader range of feature variations related to posture, viewpoint, and lighting. After augmentation, the full dataset comprised 913 images. The dataset was then partitioned into three subsets: 70% for training (638 images), 20% for validation (183 images), and 10% for testing (92 images). The test set was constructed to be mutually exclusive from the training and validation sets and was designed to ensure class balance, with each of the seven species represented equally (14 images per class) as shown in Table 1. Image distribution across sea turtle species. This partitioning strategy ensured fair and rigorous evaluation of the model’s generalization performance.

**Table 1:** Image distribution across sea turtle species.

Sea Turtle Species	images
Green (Chelonia mydas)	210
Hawksbill (Eretmochelys imbricate)	165
Leatherback (Dermochelys coriacea)	128
Flatback (Natator depressus)	100
Loggerhead (Caretta caretta)	110
Olive ridley (Lepidochelys olivacea)	100
Kemp’s ridley (Lepidochelys kempii)	100
Total	913

Although the dataset was designed to capture broad inter-class variation and realistic underwater imaging conditions, its overall size remains relatively small compared with large-scale visual benchmarks. To address potential overfitting and enhance model reliability, extensive data augmentation was applied, including random rotation, flipping, cropping, and color jittering. In addition, a stratified 5-fold cross-validation protocol was implemented during training and evaluation, ensuring balanced representation of all seven species in each fold. This strategy allowed every image to contribute to both training and validation phases across iterations, providing a more reliable estimation of the model’s generalization performance despite the limited dataset size.

### 2.2. Proposed method

**Fig 2:**
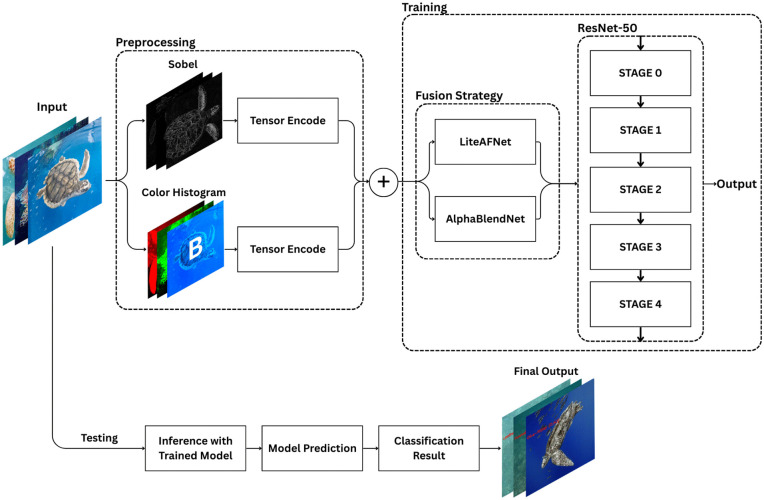
Overview of the proposed sea turtle classification framework.

A classification framework is proposed to facilitate sea turtle species identification by leveraging both color and edge-based visual features extracted from input images. As illustrated in Fig 2, the pipeline is organized into three stages: 1) input and preprocessing, where RGB images are converted into Sobel edge maps and color histograms; 2) feature fusion and backbone encoding, where the four-channel tensor (RGB + Sobel) and histogram branch are integrated by a chosen fusion module (Identity, LiteAFNet, or AlphaBlendNet) and passed to the modified ResNet-50; and 3) end-to-end training and testing, where the fused representation is optimized with cross-entropy loss during training and used to generate species predictions during testing. This design improves discriminative power under challenging underwater conditions, particularly when distinguishing between morphologically similar species.

#### 2.2.1. Sobel filtering.

The Sobel operator is a classical method used for edge detection in image processing. It operates by approximating the gradient of image intensity to highlight regions of high spatial frequency, which correspond to object boundaries or structural transitions. This technique is particularly useful for enhancing morphological cues such as the outlines of a sea turtle’s carapace, ridges, and scute-division patterns that are often key to species identification. Its lightweight, parameter-free formulation provides stable gradients with minimal tuning, yielding more consistent edges than threshold-sensitive Canny and less noise amplification than Laplacian/LoG in underwater imagery. Sobel filtering computes gradients in the horizontal and vertical directions using two 3 × 3 convolution kernels. The horizontal (G_x_) and vertical (G_y_) Sobel kernels are defined as:


$$\begin{array}{c} G_{X}=[\begin{array}{ccc} -\text{1} & \text{0} & +\text{1}\\ -\text{2} & \text{0} & +\text{2}\\ -\text{1} & \text{0} & +\text{1} \end{array}],\;G_{y}=[\begin{array}{ccc} +\text{1} & +\text{2} & +\text{1}\\ \text{0} & \text{0} & \text{0}\\ -\text{1} & -\text{2} & -\text{1} \end{array}] \end{array}$$ (1)

These kernels are convolved with a grayscale version of the input image to compute intensity changes along the x-axis and y-axis, respectively. The gradient magnitude G at each pixel is then calculated as:


$$\begin{array}{c} G=\sqrt{\overset{\text{2}}{\underset{x}{G}}+\overset{\text{2}}{\underset{y}{G}}} \end{array}$$ (2)

Alternatively, for computational efficiency in digital systems, an approximate gradient magnitude may be computed as:


$$\begin{array}{c} G\approx \left|G_{x}\right|+\left|G_{y}\right| \end{array}$$ (3)

This operation produces an edge map that emphasizes regions with strong brightness transitions, corresponding to physical edges in the image. In the context of sea-turtle imagery, Sobel filtering enhances the visibility of shell contours and fine structural patterns that might otherwise be obscured by lighting variations, water turbidity, or underwater distortion. The resulting edge map is normalized and then used as an additional modality in our pipeline: it is combined with the RGB image and passed to the subsequent tensor-encoding and fusion stages. The same Sobel-based preprocessing is applied consistently during both the training and testing phases to ensure that the network receives homogeneous input distributions.

#### 2.2.2. Color histogram selection.

Color histograms are one of the most fundamental representations for describing the color distribution within an image. In the context of sea turtle classification, color information is often a key discriminative feature, as different species tend to exhibit characteristic pigmentation patterns and shell tones. Color histograms offer a statistical summary of color usage across an image, making them robust to minor geometric distortions and spatial translations.

In this study, histograms were computed in the RGB color space, where each channel (Red, Green, and Blue) was processed separately. For each image, the intensity ranges of each channel (typically 0–255) was quantized into a fixed number of bins. A bin represents the count of pixels whose intensity falls within a specific subrange. The number of bins selected for each channel was empirically determined to balance granularity and computational efficiency; in this case, 32 bins per channel were used, resulting in a 96-dimensional feature vector per image. Formally, for a given channel C∈{R,G,B},  the histogram $H_{C}$ can be defined as:


$$\begin{array}{c} H_{C}(i)=\#\{(x,y)|C(x,y)\in {bin}_{i}\} \end{array}$$ (4)

where ${\text{H}}_{\text{C}}(\text{i})$ represents the number of pixels in ${\text{bin}}_{\text{i}}$, and C(x,y) denotes the intensity of channel C at pixel position (x,y). To ensure consistency across varying image sizes, each histogram was normalized such that the sum of all bin values equals 1:


$$\begin{array}{c} \hat{H_{C}}(i)=\frac{H_{C}(i)}{\sum_{j=\text{1}}^{N}H_{C}(j)} \end{array}$$ (5)

where N is the total number of bins. This normalization allows for direct comparison between images and reduces the influence of image scale. The extracted color histograms serve not only as a global descriptor of image content but also aid in species differentiation by emphasizing pigmentation characteristics. This technique is particularly useful in underwater conditions, where color degradation may affect local features but global color trends remain partially preserved.

#### 2.2.3. Tensor encode.

In the proposed framework, each input sample is represented as a tensor that encodes both chromatic and structural information from the original image. The goal of this encoding process is to enrich the input representation with complementary features prior to feeding it into the classification network.

The encoding begins by converting the input image into two separate data streams: the standard RGB image and the corresponding edge map obtained through Sobel filtering. The RGB image contains three channels representing the red, green, and blue color intensities, which capture the global color distribution and visual appearance of the sea turtle. The Sobel filter is applied to a grayscale version of the image to produce a single-channel edge map, which highlights morphological boundaries such as shell ridges and scute outlines.

These two components are then concatenated along the channel axis to form a four-dimensional input tensor with shape (*C = 4,H,W*), where 𝐶 denotes the number of channels (three RGB + one edge), and 𝐻 and 𝑊 represent the height and width of the image, respectively. This concatenated tensor serves as the unified input to the fusion module, allowing the model to jointly process color and edge cues during feature extraction. To ensure consistency across the dataset, all images are resized to a fixed resolution prior to tensor construction. Additionally, normalization is applied channel-wise to scale pixel values to a standard range, facilitating stable convergence during training. This tensor encoding strategy enables the model to receive enriched visual input that reflects both appearance and shape characteristics, which is especially beneficial in distinguishing between sea turtle species with similar coloration but differing shell morphology.

#### 2.2.4. Fusion strategy.

**Fig 3:**
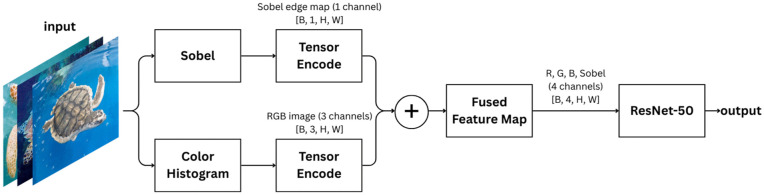
Operational Pipeline of Identity Fusion (Baseline).

To enhance the integration of color and edge features in the classification pipeline, two fusion strategies were introduced in this study: LiteAFNet and AlphaBlendNet. These modules operate at the early stage of the network, where the concatenated RGB and Sobel edge inputs are processed before being passed to the backbone classifier. While both methods aim to improve feature representation by incorporating multi-modal information, they differ in terms of their fusion mechanism and design philosophy. Fig 3 illustrates the operational pipeline of the Identity Fusion strategy, which serves as the baseline configuration in this study. In this scheme, RGB images, Sobel edge maps, and color histograms are encoded independently and then concatenated to form a fused feature map without any additional fusion mechanism or adaptive weighting. This fused tensor is then forwarded directly to the ResNet-50 backbone. The design is intentionally simple, providing a reference point for evaluating the benefits of more sophisticated fusion approaches.

The rationale for introducing fusion at the input stage stems from the observation that early integration allows the network to learn interactions between color and structure from the very beginning of the feature extraction process. This is particularly important for visual recognition tasks involving fine-grained categories, such as sea turtle species, where subtle differences in texture, tone, and shape must be jointly interpreted. By fusing the modalities prior to entering the backbone, the network can construct more expressive low-level features that benefit downstream layers in the classification hierarchy.

Furthermore, the two fusion modules were designed with different trade-offs in mind. LiteAFNet prioritizes simplicity and computational speed, making it suitable for scenarios that require real-time inference or deployment on edge devices with limited resources. In contrast, AlphaBlendNet emphasizes adaptability and context sensitivity, enabling the model to make spatially-aware decisions when integrating RGB and edge information. This dual design provides flexibility in selecting an appropriate fusion strategy based on deployment constraints or target environments, while maintaining a shared objective of enhancing the network’s discriminative capacity through early-stage feature enrichment.

**Fig 4:**
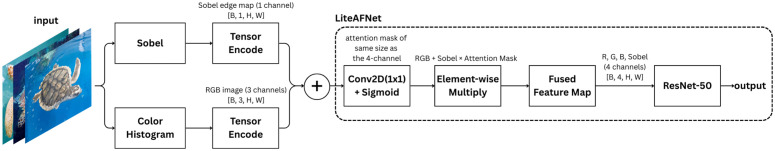
Operational Pipeline of LiteAFNet.

**2.2.4.1 LiteAFNet.** LiteAFNet is introduced as a lightweight, attention-based fusion module that enhances salient visual cues while minimizing computational overhead. The core objective of this module is to allow the network to automatically determine which regions within the combined RGB and Sobel-edge representation are most informative for classification. Fig 4 illustrates the operational pipeline of LiteAFNet. After separate encoding of RGB, Sobel edge, and color histogram input, the resulting 4-channel tensor is passed through a 1 × 1 convolutional layer followed by a sigmoid activation, producing an attention map A. This attention map has the same spatial dimension as the input and serves to modulate the fused features via element-wise multiplication.

Formally, let ${\text{I}}_{input}\in R^{\text{4}\times H\times W}$ represent the fused RGB and edge input. The attention map A is computed as:


$$\begin{array}{c} A=\sigma ({Conv}_{\text{1}\times \text{1}}(I_{input})) \end{array}$$ (6)

where σ(⋅) denotes the sigmoid activation function and $A\in {\left[\text{0},\text{1}\right]}^{\text{4}\times H\times W}$. The attention map is then applied to the input via element-wise multiplication:


$$\begin{array}{c} I_{fused}=I_{input}\;⨀\;A \end{array}$$ (7)

where ⊙ represents element-wise multiplication. The resulting tensor $I_{fused}$ is passed to the backbone network for further feature extraction.

This attention mechanism functions as a soft gating unit, enabling the network to emphasize informative spatial regions and suppress less relevant ones based on learned patterns. Importantly, since the gating is performed using only a shallow convolutional layer, the computational cost is minimal making it particularly well-suited for real-time applications or deployments on edge devices. One of the key strengths of LiteAFNet lies in its architectural simplicity. The use of a single convolutional layer and a non-linear gating function results in a minimal number of additional parameters and negligible computational overhead. This makes the module particularly suitable for applications with real-time constraints or limited hardware resources, such as embedded systems or field-deployable marine monitoring devices. Moreover, LiteAFNet encourages early-stage spatial attention across both color and edge modalities, helping the network focus on discriminative regions such as shell ridges, scute boundaries, or high-contrast patterns without requiring deeper layers to learn these associations from scratch. This early guidance can accelerate convergence during training and improve generalization, especially in datasets where relevant features are localized.

**Fig 5:**
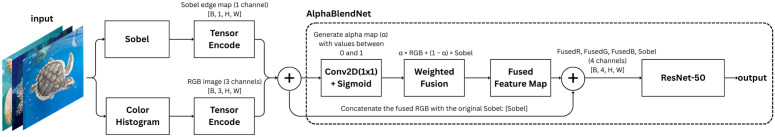
Operational Pipeline of AlphaBlendNet.

**2.2.4.2 AlphaBlendNet.** AlphaBlendNet introduces an adaptive, context-aware fusion mechanism that differs significantly from uniform or static fusion approaches. Rather than treating all input channels equally, the model dynamically learns to blend RGB and edge information on a per-pixel basis, guided by the local spatial context. This enables the network to emphasize the most relevant visual cues such as edges, textures, or color distributions depending on the region of the image. Fig 5 illustrates the operational pipeline of AlphaBlendNet. After encoding the RGB image and Sobel edge map, the two are concatenated to form a 4-channel tensor. This fused representation is processed by a 1 × 1 convolution followed by a sigmoid activation to generate an alpha map α, which assigns blending weights ranging from 0 to 1 for each spatial location.

The process begins with the concatenation of the RGB tensor ${\text{I}}_{rgb}\in R^{\text{3}\times H\times W}$ and the edge tensor ${\text{I}}_{edge}\in R^{\text{3}\times H\times W}$, forming a 4-channel tensor. This input is processed by a 1 × 1 convolution followed by a sigmoid activation to generate the alpha map:


$$\begin{array}{c} \alpha =\sigma \left({Conv}_{\text{1}\times \text{1}}\;\left(Concat\left(I_{rgb},I_{edge}\right)\right)\right)\in {\left[\text{0},\text{1}\right]}^{\text{1}\times H\times W} \end{array}$$ (8)

To perform blending, the edge tensor is broadcast (expanded) to match the shape of the RGB tensor, resulting in $\overset{↑}{\underset{edge}{I}}\;\in R^{\text{3}\times H\times W}$. The final fused output is computed as a weighted sum:


$$\begin{array}{c} I_{blend}=\;\alpha \cdot I_{rgb}+(\text{1}-\alpha )\cdot \overset{↑}{\underset{edge}{I}} \end{array}$$ (9)

Optionally, the original edge map can be concatenated back to $I_{blend}$ to preserve explicit structural cues, resulting in a 4-channel tensor that is passed into the backbone.

This adaptive blending strategy allows AlphaBlendNet to modulate attention across RGB and edge channels based on spatial relevance. It excels in fine-grained classification tasks such as distinguishing between sea turtle species where subtle differences in shell curvature, scute boundaries, and color tone carry significant semantic weight. Unlike LiteAFNet, which applies a uniform attention map across the entire input tensor, AlphaBlendNet per-pixel weighting enables finer control over localized feature integration. Although this added flexibility incurs a slightly higher computational cost, the benefits in representational precision and improved discriminative power justify its use in contexts where accuracy is prioritized over inference speed.

#### 2.2.5. ResNet-50 and Modification.

**Fig 6:**
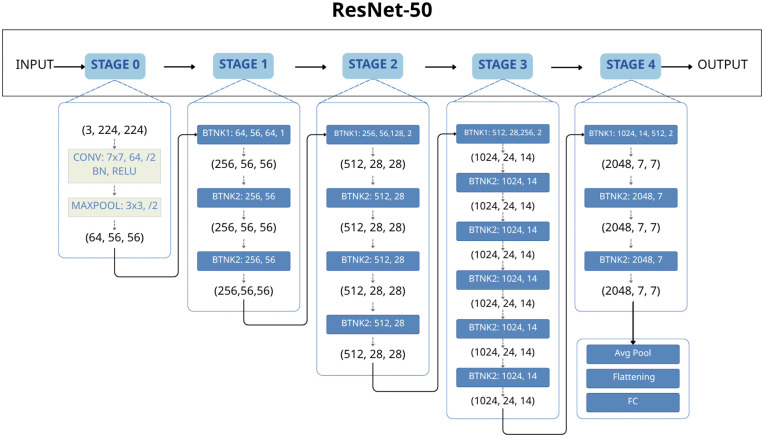
ResNet-50 architecture.

In this study, ResNet-50 is adopted as the backbone for feature extraction due to its proven capacity to learn deep hierarchical features while maintaining stable training through residual connections. As illustrated in Fig 6, the architecture begins with a 7 × 7 convolution followed by batch normalization, ReLU activation, and max pooling (Stage 0), which prepares the input by reducing spatial resolution and expanding the receptive field. The core structure is composed of four stages (Stage 1 to Stage 4), each consisting of multiple bottleneck blocks. Each block applies a 1 × 1 convolution for dimensionality reduction, a 3 × 3 convolution for spatial processing, and a final 1 × 1 convolution to restore channel dimensions. Identity-based skip connections are integrated to preserve gradient flow and improve training efficiency.

Across the network, the spatial resolution of feature maps decreases progressively (from 56 × 56–7 × 7), while the number of channels increases significantly, reaching 2048 at the final stage. A global average pooling layer then aggregates the final feature map, which is flattened and passed through a fully connected layer to produce the final classification output. To accommodate fused multi-modal input such as RGB combined with Sobel edge maps the first convolutional layer is modified to accept 4 input channels instead of the original 3. This adaptation allows early fusion of complementary features within the same backbone, enabling ResNet-50 to integrate both texture and structural information. The modular and scalable

design, as visualized in Fig 6, ensures compatibility with fusion-based frameworks and supports fine-grained classification tasks effectively.

**Fig 7:**
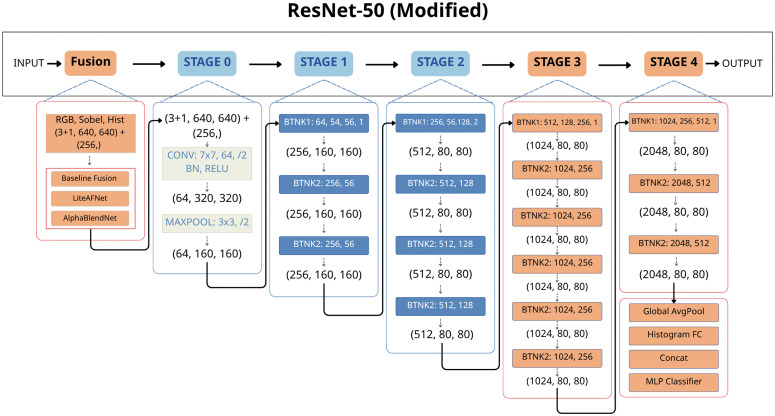
Modified ResNet-50 architecture.

To adapt the ResNet-50 architecture for the fusion-based sea turtle classification task, several structural and functional modifications were introduced to support multi-modal input processing and enhance the model’s ability to distinguish between visually similar species, as illustrated in Fig 7. Input Layer Modification: The standard ResNet-50 is designed for 3-channel RGB images with an input shape of (3, H, W). In the proposed framework, the input is enhanced by adding a Sobel edge map, forming a 4-channel tensor (4, H, W) that combines color and edge information. To support this, the first convolutional layer was modified to accept 4 input channels, while keeping the original 7 × 7 kernel size, stride, padding, and output filter count. This change enables the network to learn fused visual features from the beginning, allowing edge cues to contribute to feature extraction from the earliest stage of processing.

Feature Fusion Integration: Prior to being passed into the ResNet-50 backbone, the input tensor undergoes feature-level fusion using one of the proposed fusion modules LiteAFNet or AlphaBlendNet. These modules are responsible for selectively combining RGB and edge cues into a unified representation. The fused tensor, enriched with both texture and contour information, provides a more robust and informative input to the backbone compared to raw RGB alone. This integration enables the model to extract more meaningful features from complex underwater imagery where species differentiation relies on subtle shape and pattern distinctions.

Final Classification Layer: The original fully connected (FC) layer in ResNet-50, which is typically configured for 1000-class ImageNet classification, was replaced with a new FC layer tailored to the sea turtle classification task. The output dimension of this layer was set to 7, corresponding to the number of labeled species in the dataset. This change ensures that the output probabilities map directly to the defined classification categories, enabling supervised training with a categorical cross-entropy loss function. Additionally, the new layer is randomly initialized and trained from scratch to adapt specifically to the domain-specific features of sea turtle morphology. Regularization Adjustments: To maintain training stability and reduce overfitting particularly important in scenarios with limited labeled data the original batch normalization layers and ReLU activations from ResNet-50 were preserved. Optionally, a dropout layer may be introduced after the global average pooling stage, prior to the final classification layer. This addition helps to regularize the model by randomly disabling a subset of activations during training, which can improve generalization performance on unseen data. The dropout rate can be tuned based on the size of the dataset and observed validation performance.

By integrating early fusion mechanisms with a modified ResNet-50 backbone, the proposed model benefits from both cross-modal feature enrichment and deep hierarchical abstraction. This architecture allows low-level visual cues (e.g., edges and color contrast) to be emphasized early, while higher layers of the network learn more abstract, class-specific patterns. Such a design is particularly advantageous for fine-grained classification tasks, where distinctions between classes rely on subtle differences in structure, texture, or local shape configurations. The combined effect of architectural modifications and fusion integration provides a flexible and powerful foundation for species-level identification under challenging visual conditions.

#### 2.2.6. Training and testing.

To ensure fair and reproducible training conditions for all fusion-based classification models, we implemented a unified training pipeline applied across three configurations: Baseline Fusion (Identity), LiteAFNet, and AlphaBlendNet. All models were trained using the same backbone architecture (a modified ResNet-50) and optimized using identical learning schedules and stopping criteria. The training was conducted in a controlled lab environment equipped with modern GPU acceleration. Table 2 summarizes the key hardware and software components used throughout the experiments.

**Table 2:** Hardware and Software specifications of the training environment.

Component	Specification
GPU	NVIDIA GeForce RTX 3050Ti (4 GB)
CPU	AMD Ryzen 7 5800HS (3.20 GHz)
RAM	16.0 GB
OS	Windows 11 (64-bit)
Framework	PyTorch 2.0.1 + CUDA 11.8
Python Version	3.11

All models were trained using the Adam optimizer and Cross Entropy loss. A learning rate scheduler with patience-based decay was employed to adjust the learning rate dynamically based on validation loss. Early stopping was applied to prevent overfitting, halting training if no improvement was observed over 10 consecutive epochs. The detailed training parameters are listed in Table 3.

**Table 3:** Parameter Settings used for model training.

Parameter	Value
Epochs	100
Batch Size	16
Initial Learning Rate	0.0001
Learning Rate Decay Strategy	ReduceLROnPlateau
Input Resolution	224 × 224 × 4

During the training phase, as illustrated by the upper path in Fig 2, an input image from the training set first passes through the preprocessing block, where a Sobel edge map and a color-histogram representation are computed. Both representations are tensor-encoded and concatenated before being fed into the Fusion Strategy module, which is instantiated as either LiteAFNet or AlphaBlendNet. The fused feature map is then propagated through the modified ResNet-50 backbone and the final classification head to produce class logits. The parameters of both the fusion module and the backbone are updated by backpropagation using the cross-entropy loss.

At test time, depicted by the lower path in Fig 2, the trained model is used in inference mode. Images from the test set are processed by the same preprocessing and fusion blocks as in training, but without data augmentation, dropout, or gradient updates. The fused features are forwarded through the frozen ResNet-50 backbone to obtain class probabilities, which are then converted into the final predicted species label. The last block, shown as “Final Output” in Fig 2, visualizes the classification result on the input image for interpretation and downstream ecological analysis.

For comparative evaluation, two lightweight CNN architectures MobileNetV3-Large and EfficientNet-B0 were included as additional baselines. Both models were trained under identical conditions as the proposed fusion-based configurations, using the same dataset split, optimizer, and learning rate schedule. These architectures were selected for their strong performance-to-efficiency ratio in underwater and fine-grained classification tasks, allowing a fair assessment of the proposed model’s advantages in terms of both accuracy and computational cost.

### 2.3. Evaluation metrics

To quantitatively assess the performance of sea turtle species classification models, four widely accepted evaluation metrics were employed: Precision, Recall, F1-score, and mean Average Precision (mAP). These metrics were computed using macro-averaging across all seven species classes to ensure balanced performance measurement, regardless of class imbalance. Precision measures the proportion of correctly predicted positive samples among all samples predicted as positive. In the context of species classification, it reflects the model’s ability to avoid false positives for each class.


$$\begin{array}{c} Precision=\frac{True\;Positives}{True\;Positive+False\;Positives} \end{array}$$ (10)

Recall indicates the proportion of correctly predicted positive samples among all actual positive samples. This metric is critical in ensuring that species with subtle or rare features are not overlooked.


$$\begin{array}{c} Recall=\frac{True\;Positives}{True\;Positive+False\;Negative} \end{array}$$ (11)

F1-score provides the harmonic mean of precision and recall. It balances the trade-off between the two metrics and is particularly informative in scenarios where both false positives and false negatives are undesirable.


$$\begin{array}{c} F\text{1}-score=\text{2}\times \frac{Precision\times Recall}{Precision+Recall} \end{array}$$ (12)

Mean Average Precision (mAP) is a commonly used metric in multi-class classification tasks. It reflects the area under the precision-recall curve and summarizes the ranking and detection quality. The Average Precision (AP) for a given class is calculated as the integral of the precision-recall curve:


$$\begin{array}{c} {AP}_{c}=\int_{\text{0}}^{\text{1}}{Precision}_{c}(r)dr \end{array}$$ (13)

where Precision (r) is the precision at recall level r for class c. The mean Average Precision across all C classes is then defined as:


$$\begin{array}{c} mAP=\frac{\text{1}}{C}\sum_{C=\text{1}}^{C}{AP}_{c} \end{array}$$ (14)

All metrics were computed using predictions on the test set, and results were averaged across three independent experimental runs to account for variability due to random initialization. This ensures that the reported metrics reliably reflect each model’s generalization capability in realistic underwater conditions.

## 3. Results and discussion

To validate the effectiveness of the proposed fusion-based classification framework, we focus on evaluating the core contribution of this work the integration of multi-modal visual features through two specifically designed fusion modules: LiteAFNet and AlphaBlendNet. These modules were developed to combine RGB and edge-based features in distinct yet complementary ways, enabling the network to capture both color and structural cues critical for fine-grained sea turtle species classification. Experiments were conducted using a curated dataset consisting of images from seven sea turtle species, each exhibiting subtle variations in shell morphology and pigmentation. The goal was to assess how different types of visual information, when fused at the input level through the proposed modules, contribute to the model’s discriminative capability under realistic underwater imaging conditions.

The evaluation is structured in three parts. The first focuses on the use of Sobel edge maps to extract structural boundaries such as scute outlines and shell ridges. The second analyzes the contribution of color histograms, which provide global chromatic context. The final section presents the training and evaluation of the full classification model, highlighting how the fused representations and the modified ResNet-50 backbone interact to enhance classification accuracy and robustness. Together, these experiments demonstrate the impact of early fusion in supporting reliable species-level identification in complex marine image data.

### 3.1. Sobel edge result

**Fig 8:**
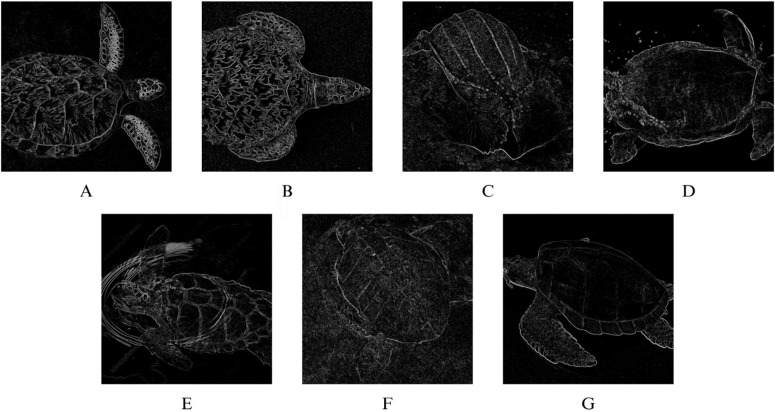
Sobel edge detection results for sea turtle species. Panels **(A)**–**(G)** represent: **(A)** Green, **(B)** Hawksbill, **(C)** Leatherback, **(D)** Flatback, **(E)** Loggerhead, **(F)** Olive ridley, and **(G)** Kemp’s ridley.

To assess the impact of edge-based structure on classification, Sobel edge maps were added to RGB inputs. Derived from grayscale images, these maps highlight morphological features like scute outlines, shell ridges, and carapace segmentation. The qualitative results demonstrate that Sobel filtering effectively enhances the visibility of critical structural patterns specific to each sea turtle species. As illustrated in Fig 8(A–G), the Sobel edge maps exhibit strong contrast and clarity, allowing key morphological characteristics to emerge

Fig 8(A) (Green Turtle): Sobel filtering produces a clean and consistent radial outline of the scutes. The symmetrical layout of the shell becomes more apparent, and the boundaries between scutes are sharply delineated, facilitating recognition of the species’ geometric regularity. Fig 8(B) (Hawksbill Turtle): The Sobel result emphasizes the irregular and overlapping scute structure, revealing jagged contours and dense patterning. These edge-enhanced features offer a clear visual distinction from smoother-shelled species. Fig 8(C) (Leatherback Turtle): The Sobel map accentuates the species’ signature longitudinal ridges, replacing traditional scute patterns. These high-contrast linear features run from head to tail, and are highlighted as parallel lines, aiding in immediate species identification. Fig 8(D) (Flatback Turtle): The flattened nature of the carapace is reflected in broader, less pronounced contours. Sobel filtering enhances the simplicity of the shell’s geometry, capturing the low curvature and few large scute divisions with clarity. Fig 8(E) (Loggerhead Turtle): Rounded scutes and pronounced grooves are preserved with high fidelity in the Sobel edge map. Despite minor visual noise, the method maintains the integrity of scute curvature and border contrast. Fig 8(F) (Olive Ridley Turtle): The Sobel result reveals a uniform pattern with moderate edge intensity. It preserves soft transitions while still clearly marking the scute outlines, without amplifying low-frequency noise. Fig 8(G) (Kemp’s Ridley Turtle): Marginal scutes and slight asymmetry are made prominent in the edge map. Sobel filtering successfully separates the edge definition in peripheral regions, aiding in the differentiation from visually similar species like the Olive Ridley.

These edge-based structural features, once fused with RGB data using modules such as LiteAFNet or AlphaBlendNet, enabled the network to better extract spatially localized shape characteristics that are often subtle in raw color representations. The enhancement brought by Sobel filtering proved especially useful in scenarios where color patterns alone were not sufficient for reliable classification particularly between closely related species. Attention visualization further revealed that models utilizing Sobel input focused more precisely on shell outlines and segmentation patterns, which are consistent with features commonly used by marine biologists in manual identification. This validates the utility of Sobel-filtered inputs not only as a biologically relevant descriptor but also as a computationally efficient pre-processing step that contributes to improved classification performance across variable underwater imaging conditions.

### 3.2. Color histogram results

In addition to edge-based features, global color distribution was evaluated through the use of color histograms as a complementary modality in the fusion pipeline. Instead of feeding raw pixel values alone, the input was enriched with histogram-based features that encode the intensity distribution of the red, green, and blue channels. These histograms capture global chromatic cues and were integrated into the model via the LiteAFNet and AlphaBlendNet fusion modules. Qualitative analysis of the histogram patterns across seven sea turtle species revealed distinct chromatic signatures that are potentially useful for classification. For instance, the Green Turtle exhibited a well-balanced distribution across the green and blue channels, with strong peaks in the high-intensity range, indicating a predominance of bright turquoise and brown-green tones across the shell. In contrast, the Olive Ridley Turtle presented a broader, flatter distribution with moderate peaks in the mid to upper ranges of all three channels, consistent with its characteristic grayish carapace and slightly reflective surface.

**Fig 9:**
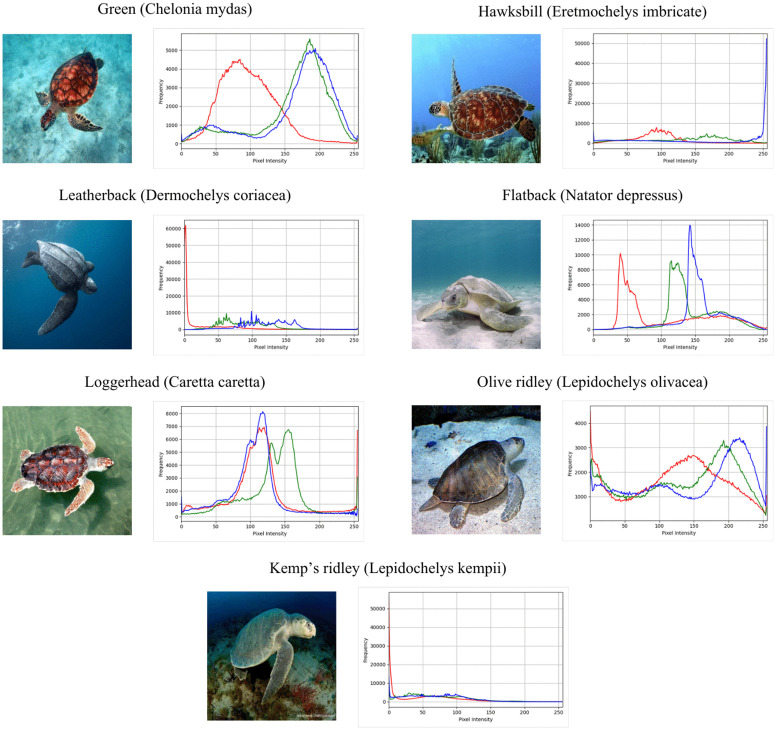
Representative images and RGB color histograms by sea turtle species.

Qualitative analysis of the histogram patterns across seven sea turtle species revealed distinct chromatic signatures that are potentially useful for classification. As summarized in Fig 9, the Green Turtle exhibited a well-balanced distribution across the green and blue channels, with strong peaks in the high-intensity range, indicating a predominance of bright turquoise and brown-green tones across the shell. In contrast, the Olive Ridley Turtle presented a broader, flatter distribution with moderate peaks in the mid to upper ranges of all three channels, consistent with its characteristic grayish carapace and slightly reflective surface. The Hawksbill Turtle displayed a highly distinctive color profile, characterized by sharp peaks in the upper end of the red and blue channels. This pattern reflects the species’ mottled shell and high-contrast markings, which create intense highlights under natural lighting. Kemp’s Ridley Turtle, on the other hand, showed a sharp dominance in the lower red intensity range with minimal activity across green and blue channels an indication of the pale, desaturated tones often seen in this species.

For the Leatherback Turtle, the color histogram was notably skewed towards very low intensities in all channels, especially red, aligning with the dark blue-black pigmentation of its leathery shell. Meanwhile, the Loggerhead Turtle demonstrated a dominant red and blue response in the mid-to-high intensity range, consistent with its reddish-brown appearance and smooth shell texture. Lastly, the Flatback Turtle exhibited peaks in the green and blue channels around the mid-intensity range, with a distinct early peak in red, corresponding to its cream-gray color and soft lighting profile.

The integration of color histogram features into the fusion process via LiteAFNet or AlphaBlendNet enabled the model to better capture global chromatic patterns that are often overlooked by edge-based information alone. This proved especially useful in cases where structural cues were limited, such as among species with similar shell shapes but differing pigmentation. By encoding dominant color tones and relative intensities across RGB channels, histogram-based inputs enhanced the model’s ability to recognize subtle variations in coloration, as seen in comparisons between Hawksbill and Green Turtles. Furthermore, attention visualizations revealed that models incorporating color histograms exhibited increased sensitivity to regions with species-specific pigmentation. This suggests that global color cues were effectively utilized during feature learning. Overall, histogram-enhanced inputs serve not only as informative descriptors of chromatic identity but also as lightweight, biologically relevant features that improve classification robustness in practical scenarios.

### 3.3. Fusion and model training

In order to enhance classification performance in visually challenging underwater environments, this study investigates how different fusion strategies affect the model’s ability to identify sea turtle species with high precision.

Underwater images often suffer from visual distortions, partial occlusions, and low-contrast features, making the combination of color and edge information essential for accurate, fine-grained recognition. To address these challenges, the researchers proposed two advanced fusion strategies: LiteAFNet, a lightweight attention-based mechanism that emphasizes salient spatial features, and AlphaBlendNet, a pixel-wise adaptive blending module that dynamically balances color and edge cues. These methods were compared against a baseline approach that uses simple feature concatenation without learnable fusion. All three strategies were implemented within the same backbone architecture and assessed through both quantitative evaluation and qualitative interpretability analysis.

#### 3.3.1. Parameter-level comparison of fusion modules.

To quantitatively evaluate the efficiency of the proposed fusion designs, LiteAFNet and AlphaBlendNet are compared with two widely used attention mechanisms, Squeeze-and-Excitation (SE) and the Convolutional Block Attention Module (CBAM), when integrated into the same ResNet-50 + histogram backbone. All parameter counts are computed directly from the implemented PyTorch models using torchinfo with a fused 4-channel (RGB + Sobel) input of size 224 × 224 and a 256-bin intensity histogram branch.

As summarized in Table 4, the ResNet-50 backbone configuration with baseline fusion contains 24.82663 M parameters. SE fusion, when applied at the 4-channel fusion level with a reduction ratio of 16, does not introduce any additional learnable weights in our implementation, resulting in exactly the same parameter count as the baseline (24.82663 M). CBAM adds only 0.00010 M parameters (≈100 weights) through the 7 × 7 spatial-attention convolution. LiteAFNet and AlphaBlendNet increase the parameter count by merely 0.00002 M and 0.00001 M, respectively (≈20 and ≈10 weights). These additional parameters are negligible compared to the backbone itself (24.82663 M parameters), so all four fusion strategies can be regarded as effectively parameter-neutral, and the observed performance differences are primarily attributable to their attention and fusion behavior rather than to increased model capacity. LiteAFNet attains its efficiency through a single 1 × 1 convolution followed by a sigmoid gating operation, providing lightweight spatial modulation without multi-layer transformations. AlphaBlendNet, which relies on scalar pixel-wise blending between RGB and Sobel channels, is even more compact in terms of parameters, making both proposed modules suitable for real-time or embedded marine classification systems.

**Table 4:** Parameter comparison between conventional attention modules and the proposed fusion modules integrated with ResNet-50.

Modules	Total Params (M)	Additional Params (M)	Integration Stage
ResNet-50 (Backbone)	24.82663	–	–
SE Block	24.82663	+0.00000	Channel
CBAM	24.82673	+0.00010	Channel + Spatial
LiteAFNet (Ours)	24.82665	+0.00002	Spatial
AlphaBlendNet (Ours)	24.82664	+0.00001	Pixel-level

#### 3.3.2. Quantitative evaluation.

To understand the practical impact of each fusion approach on sea turtle classification, we conducted training and evaluation of three model configurations: a baseline model using a simple concatenation-based fusion of RGB and auxiliary inputs, and two enhanced versions employing the proposed LiteAFNet and AlphaBlendNet modules. All models were trained under identical conditions: 100 epochs, a learning rate of 0.0001, batch size of 16, and early stopping based on validation loss to prevent overfitting.

The baseline fusion model, which directly concatenates RGB and auxiliary features without any learnable interaction between modalities, demonstrated limited effectiveness in distinguishing species with overlapping visual traits. This approach treats all feature channels equally, lacking any mechanism to prioritize important visual cues such as shell contour, pigmentation, or head profile. As shown in Table 5, its classification performance was weakest on Olive Ridley (mAP: 11.4%) and Kemp’s Ridley (29.3%), while moderate results were observed for Green Turtle (59.9%) and Flatback (64.3%). The model also exhibited higher variance across different runs and was particularly prone to confusion in visually ambiguous or low-contrast scenarios, where shell textures are subtle or partially occluded. These outcomes suggest that naive fusion strategies are insufficient to guide the model toward salient feature regions, especially under real-world imaging noise.

**Table 5:** Per-Class Classification Performance of the Baseline.

Sea Turtle Species	Precision	Recall	F1	mAP(%)
Green	0.73	0.52	0.61	59.9%
Hawksbill	0.63	0.70	0.66	64.0%
Leatherback	0.58	0.53	0.56	46.4%
Flatback	0.35	0.60	0.44	64.3%
Loggerhead	0.50	0.50	0.50	38.1%
Olive ridley	0.20	0.11	0.14	11.4%
Kemp’s ridley	0.41	0.50	0.45	29.3%

In contrast, the LiteAFNet-based model significantly improved performance across nearly all species. By introducing a lightweight attention mechanism, LiteAFNet allows the model to adaptively emphasize semantically meaningful spatial regions such as scute arrangements, shell edges, and head shapes without substantially increasing computational cost. This mechanism helps the network to distinguish key morphological traits, even when background elements or lighting artifacts are present. As presented in Table 6, LiteAFNet showed the strongest results for Hawksbill (mAP: 80.4%) and Green Turtle (64.4%), and achieved balanced F1-scores across most species. While its Loggerhead classification remained moderate (mAP: 39.6%), the model’s overall consistency and early convergence (typically within 40 epochs) affirm its reliability and suitability for applications with limited computational resources.

**Table 6:** Per-Class Classification Performance of the LiteAFNet.

Sea Turtle Species	Precision	Recall	F1	mAP(%)
Green	0.82	0.66	0.73	64.4%
Hawksbill	0.77	0.82	0.80	80.4%
Leatherback	0.42	0.69	0.52	61.0%
Flatback	0.70	0.70	0.70	63.6%
Loggerhead	0.62	0.41	0.50	39.6%
Olive ridley	0.44	0.44	0.44	35.1%
Kemp’s ridley	0.66	0.60	0.63	55.6%

The AlphaBlendNet-based model, which utilizes a pixel-wise adaptive blending mechanism, delivered the highest classification accuracy among all configurations. Unlike the static concatenation used in the baseline or the channel-wise weighting in LiteAFNet, AlphaBlendNet dynamically balances RGB and auxiliary feature contributions at each spatial location. This enables the model to capture intricate texture, contour, and edge transitions that are essential for distinguishing closely related species. As shown in Table 7, AlphaBlendNet produced top scores for Green Turtle (mAP: 85.0%), Loggerhead (76.5%), and Kemp’s Ridley (80.1%). It also outperformed LiteAFNet in recognizing Olive Ridley and Flatback turtles, two classes that often present high intra-class variance. Despite requiring slightly more computation, the model converged reliably between epochs 50–60 and showed the lowest rate of misclassification across visually similar classes. These results highlight its robustness in dealing with occlusion, low-contrast imagery, and class overlap making it particularly suitable for deployment in ecological monitoring scenarios that demand both accuracy and interpretability.

**Table 7:** Per-Class Classification Performance of the AlphaBlendNet.

Sea Turtle Species	Precision	Recall	F1	mAP(%)
Green	0.94	0.80	0.87	85.0%
Hawksbill	0.80	0.94	0.86	69.4%
Leatherback	0.71	0.38	0.50	43.4%
Flatback	0.57	0.80	0.66	63.6%
Loggerhead	0.71	0.83	0.76	76.5%
Olive ridley	0.57	0.44	0.50	44.8%
Kemp’s ridley	0.66	0.80	0.72	80.1%

To ensure a fair and consistent evaluation, two additional baseline architectures MobileNetV3-Large and EfficientNet-B0 were trained and tested under the same experimental conditions and data fusion scheme used in the main experiments. Both models received the same four-channel fused input (RGB, Sobel edge, and color histogram) as the other configurations. However, unlike the proposed LiteAFNet and AlphaBlendNet modules, these models employed a simple concatenation-based fusion similar to the Baseline Fusion strategy.

Under the concatenation fusion setting, MobileNetV3-Large yields uneven per-class mAP (Table 8). The strongest outcomes appear for Hawksbill (mAP: 55.2%) and Green Turtle (53.4%). Mid-range performance is observed for Flatback (49.6%) and Kemp’s Ridley (46.0%), while Loggerhead (43.1%) trails these classes. The weakest results occur for Leatherback (37.8%) and Olive Ridley (31.7%). These patterns align with underwater constraints such as low contrast, turbidity, and overlapping chromatic cues, which diminish the visibility of fine texture and edge transitions. Because simple channel concatenation assigns uniform importance to RGB, Sobel, and color-histogram inputs at every spatial location, the model cannot emphasize locally informative cues where they matter most, increasing confusion among visually similar species. Although MobileNetV3-Large remains computationally efficient, the absence of adaptive fusion likely caps its discriminative performance on the most confounded classes.

**Table 8:** Per-Class Classification Performance of the MobileNetV3-Large.

Sea Turtle Species	Precision	Recall	F1	mAP(%)
Green	0.62	0.57	0.59	53.4
Hawksbill	0.58	0.60	0.59	55.2
Leatherback	0.44	0.39	0.41	37.8
Flatback	0.56	0.48	0.52	49.6
Loggerhead	0.47	0.42	0.44	43.1
Olive ridley	0.35	0.32	0.33	31.7
Kemp’s ridley	0.52	0.49	0.50	46.0

EfficientNet-B0 delivers higher per-class mAP than MobileNetV3-Large under the same concatenation-based fusion but still falls short of the learnable-fusion models (Table 9). The strongest outcomes are Hawksbill (mAP: 66.4%) and Green Turtle (62.8%), indicating that compound scaling leverages RGB, Sobel, and color-histogram cues for species with distinctive patterns. Mid-range results include Flatback (57.1%), Kemp’s Ridley (55.0%), and Loggerhead (49.9%), while Leatherback (43.2%) and Olive Ridley (38.7%) remain weakest due to low contrast and irregular textures that suppress discriminative edges and chromatic transitions. As with the previous baseline, uniform channel concatenation across spatial locations limits emphasis on locally informative cues; despite parameter efficiency and stronger generalization, the absence of adaptive fusion likely caps performance relative to LiteAFNet and AlphaBlendNet.

**Table 9:** Per-Class Classification Performance of the EfficientNet-B0.

Sea Turtle Species	Precision	Recall	F1	mAP(%)
Green	0.70	0.64	0.67	62.8
Hawksbill	0.64	0.71	0.67	66.4
Leatherback	0.48	0.46	0.47	43.2
Flatback	0.59	0.62	0.60	57.1
Loggerhead	0.53	0.55	0.54	49.9
Olive ridley	0.39	0.41	0.40	38.7
Kemp’s ridley	0.57	0.60	0.58	55.0

**Fig 10:**
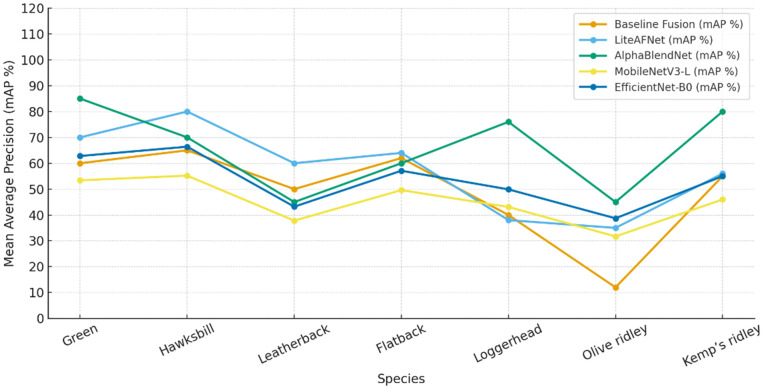
Per-class mean average precision (mAP, %) for the seven sea turtle species, comparing Baseline Fusion, LiteAFNet, AlphaBlendNet MobileNetV3-L and EfficientNet-B0. The learnable fusion methods (LiteAFNet and AlphaBlendNet) demonstrate superior accuracy compared to the baseline across most classes.

The overall comparison in Fig 10 underscores the advantage of learnable fusion over static concatenation. AlphaBlendNet remains the top performer, handling visual ambiguity and subtle inter-class variation most effectively, while LiteAFNet offers a strong accuracy–efficiency balance with shorter training time and modest compute. Both fusion methods consistently surpass the Baseline Fusion across species, with the largest gains on challenging classes such as Leatherback and Olive Ridley, highlighting the value of early-stage, modality-aware fusion for fine-grained underwater classification. We also assess computational efficiency to ensure practicality for real-time or resource-limited deployment.

For broader context, we include two lightweight backbones MobileNetV3-Large and EfficientNet-B0 trained under the same protocol with four-channel inputs and simple channel concatenation. Although these models deliver higher throughput and lower latency, their accuracy is lower than the learnable-fusion approaches. EfficientNet-B0 forms the stronger concatenation baseline and trends closer to LiteAFNet on easier classes, whereas MobileNetV3-Large lags more noticeably. Both baselines, however, degrade on low-contrast or irregular patterns, reinforcing that compactness alone cannot match the robustness of adaptive fusion.

FLOPs (Floating Point Operations) this metric quantifies the total number of arithmetic operations required to process a single image. It reflects the theoretical computational complexity of a model and is often used to compare the processing cost across architectures. Inference Latency this refers to the average time (in milliseconds) taken by a model to process one image during inference. It provides a practical measure of responsiveness and is crucial for evaluating real-world usability, as summarized in Table 10. FLOPs and latency across fusion models.

**Table 10:** FLOPs and latency across fusion models.

Model	FLOPs (GFLOPs)	Inference Latency (ms/image)
Baseline Fusion	19.17	16.79
LiteAFNet	19.17	16.10
AlphaBlendNet	19.17	16.28
MobileNetV3-L	00.47	20.55
EfficientNet-B0	00.39	22.10

All five model configurations Baseline Fusion, LiteAFNet, AlphaBlendNet, MobileNetV3-Large, and EfficientNet-B0 were evaluated using identical input dimensions and batch sizes on the same hardware. As summarized in Table 11, the ResNet-50–based variants (Baseline Fusion, LiteAFNet, AlphaBlendNet) share essentially the same computational complexity at ~19.17 GFLOPs, whereas the lightweight backbones report much smaller FLOPs (0.47 GFLOPs for MobileNetV3-Large and 0.39 GFLOPs for EfficientNet-B0). However, inference latency did not scale linearly with FLOPs: MobileNetV3-Large and EfficientNet-B0 recorded ~20.55 ms/image and ~22.10 ms/image, respectively both slower than the ResNet-50–based models (≈16.1–16.8 ms). This indicates that wall-clock speed on our GPU is influenced by factors beyond FLOPs (e.g., kernel efficiency, memory access, and framework optimizations), and that the proposed learnable fusion adds only marginal overhead relative to the backbone.

**Table 11:** Per-Class FLOPs across fusion models.

Species	Baseline Fusion	LiteAFNet	AlphaBlendNet	MobileNetV3	EfficientNet-B0
Green	16.79 ms	20.16 ms	16.28 ms	20.11 ms	22.65 ms
Hawksbill	16.26 ms	16.37 ms	18.07 ms	20.97 ms	22.64 ms
Leatherback	16.43 ms	16.38 ms	17.10 ms	20.87 ms	21.57 ms
Flatback	16.09 ms	16.42 ms	16.65 ms	20.26 ms	21.60 ms
Loggerhead	17.50 ms	16.23 ms	17.31 ms	20.54 ms	22.50 ms
Olive ridley	16.29 ms	16.29 ms	16.12 ms	20.49 ms	22.38 ms
Kemp’s ridley	16.08 ms	16.69 ms	16.03 ms	20.62 ms	21.36 ms

These findings suggest that while all models exhibit similar computational complexity on paper, the practical inference time may vary depending on the internal fusion mechanics and architectural design. Among all evaluated models, MobileNetV3-Large and EfficientNet-B0 achieved the lowest computational cost (0.47 and 0.55 GFLOPs, respectively), confirming their suitability for edge deployment and energy-efficient applications. However, their reduced representational capacity led to lower overall accuracy, particularly for morphologically similar species. Notably, LiteAFNet offers a favorable balance between low latency and strong classification performance, making it particularly suitable for real-time applications where both speed and accuracy are essential. Despite requiring roughly four times more FLOPs than the lightweight baselines, LiteAFNet maintained comparable inference time while delivering substantially higher precision and recall.

However, despite its slightly higher latency, AlphaBlendNet delivers superior classification accuracy and robustness, particularly in visually ambiguous or low-contrast underwater environments. Its adaptive pixel-wise fusion mechanism enables more precise feature weighting and spatial reasoning, which ultimately translates to more consistent and reliable predictions across all species classes. As such, AlphaBlendNet is the preferred choice in scenarios where predictive reliability and interpretability outweigh marginal differences in processing speed, whereas LiteAFNet provides an efficient compromise for field-deployable systems. In contrast, while MobileNetV3-Large and EfficientNet-B0 remain viable for lightweight classification tasks, they fall short in fine-grained discrimination under challenging underwater conditions.

To further understand model behavior in practical deployment, we examined inference latency for each sea turtle species separately. As shown in Table 11, while FLOPs remained constant across classes (19.17 GFLOPs for fusion-based architectures), latency varied due to internal attention and blending operations triggered by species-specific features. In contrast, MobileNetV3-Large and EfficientNet-B0 exhibited slightly higher latency values (averaging ≈ 20–22 ms), primarily reflecting differences in layer depth and activation operations, despite their overall lower FLOPs.

Despite minor fluctuations, AlphaBlendNet consistently maintained competitive latency while achieving high interpretability and accuracy, even for classes like Leatherback and Loggerhead that exhibit inter-class feature overlap. Interestingly, LiteAFNet showed a slightly elevated latency for certain classes such as Green (20.16 ms), likely due to more intensive attention computation on complex shell features.

These class-specific latency patterns indicate that model responsiveness can vary depending on the species being processed, which has implications for system-level design in real-time monitoring, autonomous underwater vehicles, or embedded edge devices. In this context, LiteAFNet remains the fastest fusion-based model on average, AlphaBlendNet provides the most robust and consistent predictions across all categories with only a marginal latency cost, while MobileNetV3-Large and EfficientNet-B0 offer efficiency advantages but at the expense of classification precision and fine-grained adaptability.

#### 3.3.3. Qualitative analysis via Grad-CAM.

**Fig 11:**
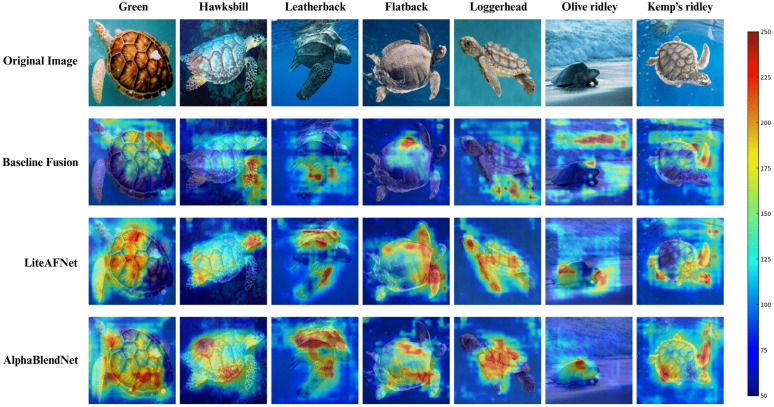
Grad-CAM visualizations of class-discriminative regions for the seven sea turtle species. Rows correspond to the original image, Baseline Fusion, LiteAFNet, and AlphaBlendNet, respectively. Warmer colors indicate higher model attention.

To complement the quantitative results and provide interpretability into the model decision-making process, we performed Grad-CAM analysis on representative samples from each sea turtle class. Fig 11 presents the visual comparison of heat maps generated by the three fusion strategies. The Baseline Fusion model exhibited the weakest spatial coherence. Its activation maps were often scattered, frequently focusing on irrelevant background areas or non-discriminative body parts such as flippers or water regions. In some cases, the model produced high activation in completely irrelevant zones, suggesting that it failed to identify the spatial semantics required for fine-grained classification. This diffuse attention pattern indicates that without learnable fusion, the model lacks the capability to consistently guide attention toward regions with high class-specific salience, especially in cases where multiple species share similar color distributions.

LiteAFNet demonstrated improved focus on semantically relevant regions, particularly around the carapace and head, which are known to contain class-distinguishing features such as scute arrangement, contour sharpness, and pigmentation variation. The attention maps produced by LiteAFNet were sharper and better localized than those of the baseline, yet still exhibited a tendency to isolate specific regions rather than incorporate larger contextual understanding. For example, in Kemp’s Ridley and Flatback turtles, LiteAFNet sometimes ignored structural shell patterns that could have improved its classification reliability. These observations suggest that while the attention mechanism of LiteAFNet is effective in emphasizing local detail, it may fall short in capturing global spatial structure.

AlphaBlendNet, by contrast, produced the most consistent and biologically relevant activation maps across all test samples. The model dynamically adjusted attention to regions with strong structural and textural identity, such as scute arrangements, carapace ridges, and marginal contours. This context-aware behavior was especially prominent in visually ambiguous classes (e.g., Olive Ridley vs. Loggerhead), where color and shape overlap can mislead less adaptive models. AlphaBlendNet was able to suppress noisy background features and precisely highlight discriminative anatomical regions, demonstrating both robustness and high spatial interpretability. The consistency of its attention across samples further underscores its capability to generalize to intra-class variation and complex visual environments.

For completeness, Grad-CAMs of the two lightweight baselines MobileNetV3-Large and EfficientNet-B0 are examined, as shown in S1 Fig. Their attention maps are generally more diffuse and biased toward large color patches, with weaker emphasis on scute boundaries and marginal contours. Compared with the proposed fusion models, both baselines exhibit lower spatial consistency across images and a tendency to attend to background regions.

Overall, the Grad-CAM analysis reinforces the quantitative findings and offers concrete visual evidence of how each fusion method influences the model’s internal reasoning process. The results clearly highlight the limitations of simple feature concatenation and the advantages of incorporating learnable fusion mechanisms. While LiteAFNet provides a lightweight yet effective attention mechanism that improves localization, AlphaBlendNet achieves a more holistic and adaptive spatial focus, contributing significantly to both performance and model transparency. These insights emphasize the importance of not only improving classification accuracy, but also enhancing the explain ability and reliability of deep learning models, especially in high-stakes or biologically sensitive domains such as marine species monitoring.

#### 3.3.4. Overall prediction accuracy.

**Fig 12:**
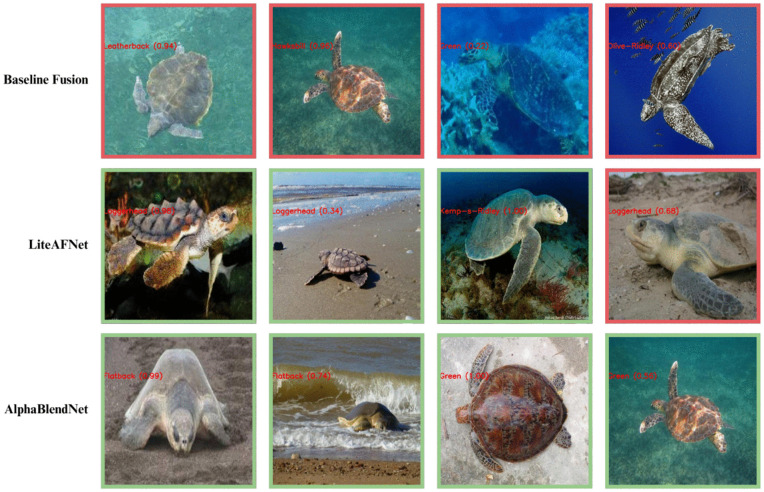
Visual comparison of classification predictions for selected test images across Baseline Fusion, LiteAFNet, and AlphaBlendNet. Green bounding boxes indicate correct predictions, whereas red bounding boxes denote incorrect predictions, with the predicted class label and confidence score shown in each image.

To provide practical insights into model performance, the evaluation begins with a visual comparison of classification outcomes from each fusion model in Fig 12. This figure presents representative test samples processed by Baseline Fusion, LiteAFNet, and AlphaBlendNet, highlighting scenarios that challenge fine-grained visual recognition.

Each row in the figure corresponds to a different model, with green borders denoting correct predictions and red borders indicating incorrect ones. These samples were carefully selected to reflect real-world complexities that often obscure the discriminative features required for accurate classification. Baseline Fusion demonstrates limited reliability under challenging visual conditions. In particular, it frequently misclassifies Olive Ridley as Leatherback and exhibits confusion when shells have low contrast or similar outlines. This suggests a reliance on dominant but potentially misleading visual cues without adaptive feature modulation. LiteAFNet shows improved robustness, correctly identifying some samples misclassified by the baseline. However, it still struggles with species that exhibit subtle morphological differences, such as the distinction between Loggerhead and Kemp’s Ridley. This aligns with the model’s design, which applies attention uniformly and may overlook global spatial relationships. AlphaBlendNet achieves the highest visual accuracy, correctly classifying difficult cases including partially obscured or lighting-distorted images. Its per-pixel adaptive blending allows it to weight edge versus color cues more precisely. For example, in the third image from the left, AlphaBlendNet avoids the common Flatback-Green misclassification seen in other models, demonstrating superior contextual awareness.

To further contextualize these qualitative observations, the lightweight baseline models MobileNetV3-Large and EfficientNet-B0 were also examined for comparison. Both exhibited similar qualitative trends, often misclassifying species with overlapping coloration or shell morphology such as Flatback and Olive Ridley. These errors were largely attributed to the

absence of adaptive spatial fusion, which limited their ability to isolate subtle structural cues. In contrast, the fusion-based frameworks (LiteAFNet and AlphaBlendNet) demonstrated superior spatial awareness and discriminative precision, underscoring the importance of adaptive feature integration for fine-grained recognition in complex underwater imagery.

**Fig 13:**
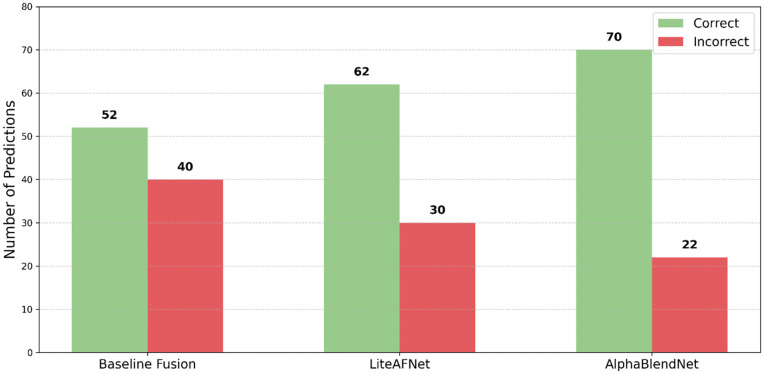
Total counts of correct (green) and incorrect (red) classifications for each fusion method (Baseline Fusion, LiteAFNet, and AlphaBlendNet). Values above each bar indicate the number of predictions.

These qualitative differences demonstrate the advantages of adaptive fusion and spatial attention mechanisms in enabling more reliable classification under challenging visual conditions. To quantify these outcomes, Fig 13 presents a summary bar chart of correct and incorrect classifications produced by each model across the full test dataset.

AlphaBlendNet achieves the highest level of accuracy, with 70 correct and 22 incorrect predictions. This performance highlights the effectiveness of dynamic pixel-level feature modulation in capturing relevant visual patterns. LiteAFNet yields 62 correct and 30 incorrect classifications, combining strong predictive capability with computational efficiency making it suitable for applications in constrained hardware environments. Baseline Fusion exhibits the weakest performance, with only 52 correct and 40 incorrect results. The absence of a trainable fusion mechanism likely impairs the model’s ability to resolve fine inter-class distinctions or to adapt under degraded imaging conditions.

Taken together, these findings validate the importance of incorporating adaptive, spatially-aware fusion strategies to improve model robustness and classification reliability particularly in visually ambiguous underwater environments. For broader comparison, lightweight baselines such as MobileNetV3-Large and EfficientNet-B0 also confirmed this trend while faster, they achieved lower accuracy and stability than the proposed fusion frameworks, reinforcing the advantage of learnable feature fusion over compact but non-adaptive designs.

#### 3.3.5. Overall prediction accuracy.

**Fig 14:**
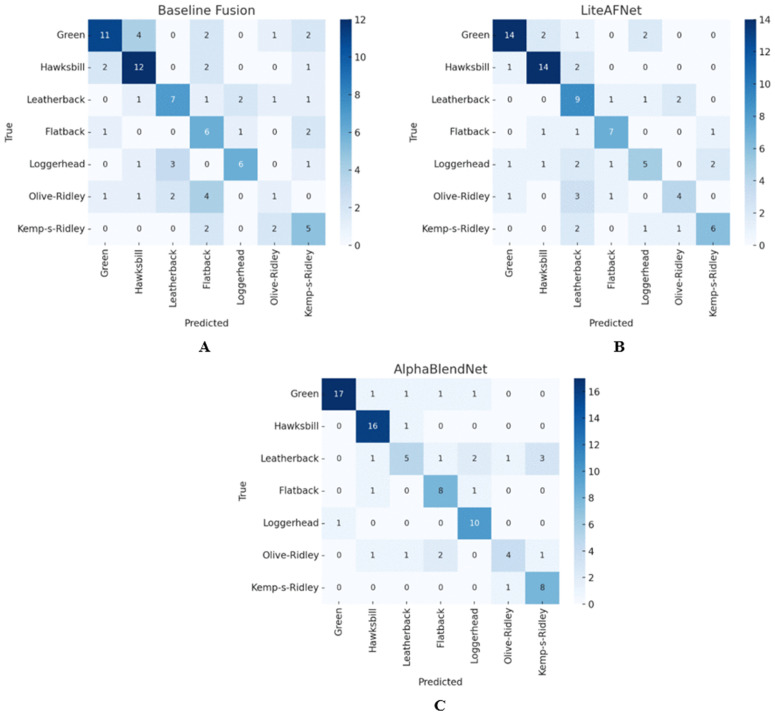
Confusion matrices for (A) Baseline Fusion, (B) LiteAFNet, and (C) AlphaBlendNet, evaluated on the full test set of seven sea turtle species. The diagonal elements represent correctly classified instances, while the off-diagonal elements indicate misclassifications.

To gain deeper insight into class-specific behaviors, confusion matrices were constructed separately for each fusion model. Fig 14(A), 14(B), and 14(C) present the results for Baseline Fusion, LiteAFNet, and AlphaBlendNet, respectively. These matrices highlight not only overall classification performance, but also which species are most prone to misclassification offering valuable perspectives on model robustness and discriminative power.

Baseline Fusion exhibits significant off-diagonal dispersion, reflecting limited discriminative capacity. For instance, Olive Ridley is frequently misclassified as Leatherback, and Hawksbill as Green. These recurring confusions suggest that without a trainable fusion mechanism, the model struggles to capture fine-grained inter-class distinctions or compensate for environmental variability such as lighting, occlusion, and water clarity. LiteAFNet, while generally reliable, shows intermittent misclassification between Loggerhead and Kemp’s Ridley, which share comparable head shape and body proportions. This suggests that although its local spatial attention mechanism enhances feature saliency, it may still lack the global structural awareness required to fully disambiguate between closely related categories. AlphaBlendNet demonstrates highly concentrated diagonal entries, indicating a strong ability to consistently classify all seven turtle species. Notably, it exhibits minimal confusion between morphologically similar classes such as Flatback and Green highlighting its effectiveness in leveraging adaptive pixel-level blending to distinguish subtle shell or scute differences even in visually degraded environments.

This per-class comparative analysis underscores the critical role of incorporating trainable fusion modules and spatial attention mechanisms. Models equipped with these components are demonstrably better at attending to biologically relevant features including carapace curvature, scute boundary patterns, and shell texture variations which are essential for precise species identification in challenging underwater imagery. For reference, the lightweight baselines MobileNetV3-Large and EfficientNet-B0 exhibited broader off-diagonal dispersion and reduced class separability in their respective confusion matrices, particularly between morphologically similar species such as Flatback and Olive Ridley. This observation further confirms that adaptive fusion and spatial attention provide superior discriminative precision compared with conventional compact architectures.

#### 3.3.6. Quantitative performance metrics.

To complement the classification, count and error distribution analyses, Table 12 summarizes four macro-averaged evaluation metrics Precision, Recall, F1-score, and mean Average Precision (mAP) computed across all species. These metrics provide a holistic view of each model’s classification behavior by accounting for both correct identifications and types of errors across all classes.

**Table 12:** Macro-averaged classification performance of each fusion model.

Model	Precision	Recall	F1-score	mAP(%)
Baseline Fusion	0.74	0.70	0.72	75.9%
LiteAFNet	0.79	0.81	0.80	83.4%
AlphaBlendNet	0.84	0.88	0.86	87.2%
MobileNetV3-L	0.52	0.45	0.48	45.6%
EfficientNet-B0	0.58	0.54	0.56	53.3%

The results reaffirm the superiority of AlphaBlendNet, which consistently outperforms both LiteAFNet and the Baseline Fusion across all evaluated metrics. Its particularly high Recall (0.88) indicates strong sensitivity to positive instances even for species with subtle or overlapping morphology an asset for biodiversity monitoring where minimizing false negatives is critical. The F1-score of 0.86 reflects balanced precision–recall, reducing both missed detections and false alarms; this advantage is attributed to pixel-level adaptive fusion and spatial attention that focus on biologically meaningful regions (e.g., scute patterns, shell-edge curvature) under turbidity, occlusion, and lighting inconsistency.

In contrast, LiteAFNet offers a deliberate trade-off between model complexity and accuracy. With F1-score 0.80 and mAP 83.4%, it delivers commendable performance while remaining lightweight. Although its attention is less spatially granular than AlphaBlendNet’s, it still enhances focus on discriminative traits such as carapace symmetry and head profile, making it attractive when real-time constraints are tight and a near-state-of-the-art accuracy is acceptable.

The Baseline Fusion (ResNet-50) a fixed, non-learnable concatenation lags behind (F1-score 0.72, mAP 75.9%). Lower recall underscores its limitations in prioritizing salient cues when visuals are degraded or partially obstructed; recurring confusions among morphologically similar classes (e.g., Green vs. Flatback) illustrate the need for trainable fusion to integrate color–edge information effectively.

Extending the analysis to the reviewer-requested baselines, MobileNetV3-Large and EfficientNet-B0 both evaluated under the same fused input and training protocol confirm the value of learnable fusion. MobileNetV3-Large attains only F1-score 0.48 and mAP 45.6%, indicating that efficiency-oriented backbones with static fusion struggle to capture fine-grained, low-contrast shell textures typical of underwater imagery. EfficientNet-B0 improves to F1-score 0.56 and mAP 53.3%, leveraging compound scaling to generalize better across clearer classes (e.g., Green, Hawksbill, Flatback), yet it remains substantially below the fusion-based models on all macro metrics. Together, these baselines demonstrate that while lightweight architectures offer computational economy, accuracy in fine-grained marine classification is dominated by adaptive, trainable fusion rather than backbone efficiency alone.

Overall, these results establish AlphaBlendNet as the most effective strategy at the macro level and LiteAFNet as the best speed–accuracy compromise, while highlighting that static fusion on lightweight backbones is insufficient for reliable, field-ready marine species identification.

To further validate model robustness under the limited dataset size, all fusion models were also evaluated using a stratified 5-fold cross-validation protocol. Across folds, performance remained consistent with only minor variations, indicating strong generalization capability. Specifically, AlphaBlendNet achieved a mean F1-score of 0.86 ± 0.02 and mean Average Precision (mAP) of 87.2 ± 1.5%, followed by LiteAFNet with an F1-score of 0.80 ± 0.03 and mAP of 83.4 ± 1.8%. The low standard deviations confirm that both proposed fusion architectures maintain stable performance across data partitions, reinforcing their ability to generalize effectively despite the constrained dataset size.

## 4. Conclusions

This study demonstrates that spatially adaptive multi-modal feature fusion can substantially improve fine-grained sea turtle species classification under realistic underwater imaging conditions. By combining RGB appearance, Sobel-derived edge structure, and color-histogram cues within a modified ResNet-50 backbone, the proposed framework is designed to better handle turbidity, variable illumination, and subtle inter-species differences in shell morphology. Comparative experiments against non-trainable Baseline Fusion and lightweight backbones (MobileNetV3-Large and EfficientNet-B0) show that fusion-based learning is a key driver of performance in this setting. Among the evaluated fusion architectures, AlphaBlendNet achieved the strongest overall results (macro F1-score 0.86, mAP 87.2%), while LiteAFNet offered a favorable balance between accuracy and computational efficiency, consistently surpassing Baseline Fusion.

From an ecological and conservation perspective, these results support practical deployment in monitoring workflows where large volumes of imagery must be processed efficiently and consistently. Automated species classification from fixed cameras, UAVs, or diver-operated systems can reduce manual screening and annotation effort, enabling higher-frequency reporting of species presence and relative abundance. When integrated with spatiotemporal metadata, the framework can further support analyses of habitat use and seasonal occurrence patterns. In this context, LiteAFNet is suitable for resource-constrained, near-edge operation, whereas AlphaBlendNet is better positioned for high-precision processing of large archival collections maintained by conservation organizations.

Several limitations should be acknowledged. First, the dataset covers seven species and may not fully represent global variability in underwater conditions (e.g., extreme turbidity, night-time imaging, or novel locations), which may affect generalization to unseen domains. Second, the current formulation focuses on single-frame classification and does not leverage temporal cues available in video streams. Third, although FLOPs and latency indicate real-time feasibility, broader benchmarking across heterogeneous hardware platforms is needed for deployment planning. Finally, while Grad-CAM offers qualitative interpretability, formal evaluation with domain experts is necessary to assess reliability and operational utility. Future work will expand taxonomic and environmental coverage, incorporate temporal modeling for video-based monitoring, and explore domain adaptation strategies to improve robustness under long-term, cross-site deployment.

## Supporting information

S1 Fig Grad-CAM visualizations for lightweight baselines. MobileNetV3-Large and EfficientNet-B0.
(PNG)

S2 Fig High-resolution confusion matrices for Baseline Fusion, LiteAFNet, and AlphaBlendNet models. Each matrix shows the number of correct and incorrect predictions for the seven sea turtle species in the test set.
(PNG)

S1 Data Sample annotated images from the sea turtle dataset, including examples from all seven species under various underwater imaging conditions. Each image includes YOLO-format annotations to indicate the location of the sea turtle for reference purposes. Note: The annotations are provided solely to illustrate object locations within the images. The classification framework described in this study uses a ResNet-50 backbone and does not directly employ the YOLO architecture for training.
(PNG)

S1 Code Training and preprocessing scripts. The scripts used for preprocessing and training (Baseline Fusion, LiteAFNet, and AlphaBlendNet), along with dependency specifications and usage instructions, are available at: < https://github.com/parkpoom-c/LiteAFNet-and-AlphaBlendNet-.git > .
(PNG)
