# Supplementary figures and images for: A deep learning-based fusion framework for robust fine-grained classification of sea turtles in support of marine biodiversity
Source: PLoS One. 2026 Jun 9;21(6):e0344942. doi: 10.1371/journal.pone.0344942 (PMC13249215; doi:10.1371/journal.pone.0344942)

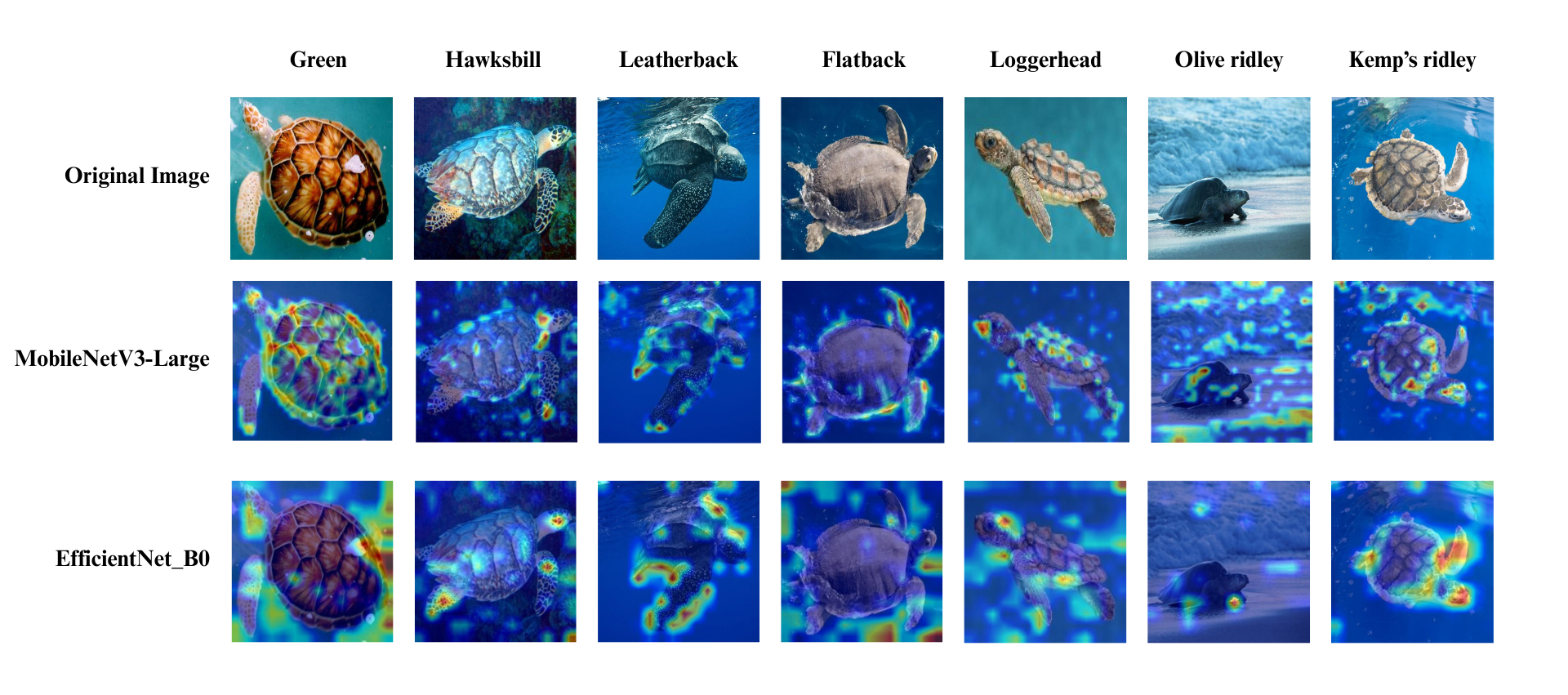

Supplement: S1 Fig — MobileNetV3-Large and EfficientNet-B0. (PNG) [file pone.0344942.s001.png]

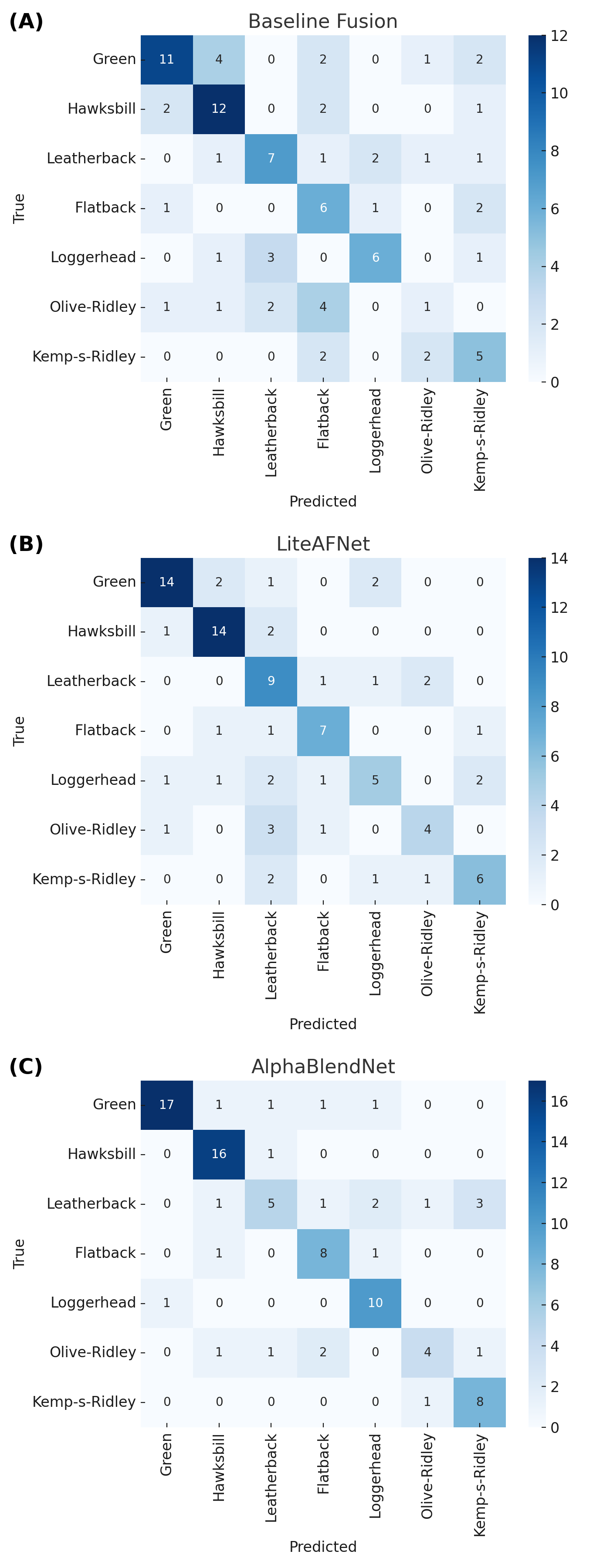

Supplement: S2 Fig — Each matrix shows the number of correct and incorrect predictions for the seven sea turtle species in the test set. (PNG) [file pone.0344942.s002.png]
