# Supplementary material for: A deep learning-based fusion framework for robust fine-grained classification of sea turtles in support of marine biodiversity
Source: PLoS One. 2026 Jun 9;21(6):e0344942. doi: 10.1371/journal.pone.0344942 (PMC13249215; doi:10.1371/journal.pone.0344942)
Supplement: Attachment — Submitted filename: Response to Reviewers.pdf [file pone.0344942.s006.pdf]

# Response to Reviewers

**Manuscript Title:** A Deep Learning-Based Fusion Framework for Robust Fine-Grained Classification of Sea Turtles in Support of Marine Biodiversity
**Journal:** PLOS ONE
**Date:** [10/24/2025]

Dear Academic Editor and Reviewer,

We thank you for your thoughtful and constructive feedback. We have revised the manuscript accordingly and believe the changes substantially improve clarity, rigor, and reproducibility. Below we respond point-by-point; all edits are marked in the Revised Manuscript with Track Changes.

# Reviewer #1

**Overall assessment**
"The manuscript proposes a deep learning-based multimodal fusion framework (RGB + Sobel) with two custom modules (LiteAFNet, AlphaBlendNet) and a modified ResNet-50; both modules improve performance. Minor revision recommended."
**Response:** We appreciate the positive evaluation and address each item below.

**1) Parameter-level comparisons for LiteAFNet and AlphaBlendNet**

**Comment:** Include parameter-level comparisons with similar attention/fusion modules (e.g., parameter count).

**Response (revised):** Completed. We added a focused subsection and a summary table that report Total parameters of the end-to-end model and Additional parameters introduced by each module under the same ResNet-50 backbone, together with the integration stage of each module. Specifically, the baseline ResNet-50 totals 23.5M params; integrating SE and CBAM yields totals of 26.02M and 26.62M, with module-only additions of +0.12M and +0.18M, respectively. In contrast, our LiteAFNet and AlphaBlendNet keep the model totals at ≈23.52M, introducing only +0.00002M and +0.00003M parameters, respectively i.e., a negligible overhead while still enabling spatial/pixel-level adaptivity. Integration stages are clarified in the table (SE: channel; CBAM: channel+spatial; LiteAFNet: spatial; AlphaBlendNet: pixel-level).

**Change in manuscript:** Added Section "Parameter-Level Comparison of Fusion Modules" and Table 2: Parameter comparison between conventional attention modules and the proposed fusion modules integrated with ResNet-50 (Total Params, Additional Params, Integration Stage); minor cross-references added in Methods.

## 2) Dataset size and generalization

**Comment:** The dataset is relatively small; expand or validate on additional datasets to demonstrate generalizability.

**Response:** Addressed. We explicitly acknowledge the limited dataset size and strengthened the validation in two ways that are now described in the manuscript:

1. **Extensive data augmentation** to mitigate overfitting and improve reliability—random rotation, flipping, cropping, and color jittering—applied consistently during training.
2. **Stratified 5-fold cross-validation** during training and evaluation, ensuring a balanced representation of all seven species in every fold. This protocol lets each image contribute to both training and validation across iterations and yields a more reliable estimate of generalization.

Across folds, performance remained stable with only minor variations, indicating strong generalization. AlphaBlendNet achieved F1 = 0.86 ± 0.02 and mAP = 87.2% ± 1.5%, followed by LiteAFNet with F1 = 0.80 ± 0.03 and mAP = 83.4% ± 1.8%. The low standard deviations confirm that both proposed fusion architectures maintain consistent performance across data partitions despite the constrained dataset size.

**Change in manuscript:** Added/expanded text in Data Collection (augmentation and CV protocol) and Quantitative Performance Metrics (5-fold CV results with mean ± SD).

## 3) Typographical error in Training (Table 3)

**Comment:** There appears to be a typographical error. Please provide the correct training parameters.

**Response:** Corrected. We have updated the training configuration and renumbered the table due to new material added in the Methods. The Parameter Settings are now:

- **Epochs:** 100
- **Batch size:** 16
- **Initial learning rate:** 0.0001
- **Learning-rate schedule:** ReduceLROnPlateau
- **Input resolution:** 224 × 224 × 4 (RGB + Sobel)

All in-text citations that previously referred to the old training table were updated to the new numbering.

**Change in manuscript:** Replaced the training table with Table 4. Parameter Settings used for model training.

## 4) Additional comparisons with commonly used models

**Comment:** Please include more widely used models to strengthen the evaluation.

**Response:** Completed. We integrated two widely adopted lightweight classifiers—MobileNetV3-Large and EfficientNet-B0—and trained/evaluated them under the same experimental protocol as our methods (identical training settings in Table 4 and the same input-fusion pipeline used in the main experiments). We report per-class and macro metrics and analyze accuracy–efficiency trade-offs.

- **New per-class results.**
  - Table 9 (MobileNetV3-Large): per-class Precision/Recall/F1/mAP; macro F1 = 0.48 and mAP = 45.6%.
  - Table 10 (EfficientNet-B0): per-class Precision/Recall/F1/mAP; macro F1 = 0.56 and mAP = 53.3%—higher than MobileNetV3-L across most species, but still below our learnable fusion modules.
  - Figure 9: per-class mAP (%) curves for the seven species now compare Baseline Fusion, LiteAFNet, AlphaBlendNet, MobileNetV3-L, and EfficientNet-B0, showing the consistent advantage of LiteAFNet/AlphaBlendNet over all baselines.
- **Efficiency analysis.**
  - Table 11 (FLOPs & latency): MobileNetV3-L = 0.47 GFLOPs / 20.55 ms, EfficientNet-B0 = 0.39 GFLOPs / 22.10 ms (single-image inference).
  - Table 12 (per-class latency): per-species inference times reported for all models; MobileNetV3-L and EfficientNet-B0 remain in the ~20–22 ms range across classes.
- **Macro-averaged summary.**
  - Table 13 now includes both new models alongside the existing methods:
    - MobileNetV3-L: Precision 0.52, Recall 0.45, F1 = 0.48, mAP = 45.6%.
    - EfficientNet-B0: Precision 0.58, Recall 0.54, F1 = 0.56, mAP = 53.3%.
    - Both trail our LiteAFNet (F1 = 0.80, mAP = 83.4%) and AlphaBlendNet (F1 = 0.86, mAP = 87.2%), confirming that learnable fusion outperforms simple concatenation-based baselines under realistic underwater conditions.

**Change in manuscript:** Added Table 9 and Table 10 (per-class metrics); updated Figure 9 (per-class mAP curves), Table 11 (FLOPs & latency), Table 12 (per-class latency), and Table 13 (macro-averaged metrics). Corresponding narrative updates were made in Results and Discussion to incorporate MobileNetV3-Large and EfficientNet-B0.

# Journal Requirements

- **Reference list audit:** We reviewed all references, added missing DOIs, corrected metadata, and replaced unverifiable entries with current, citable sources. The revised bibliography reflects these updates.
- **Figure 8 cross-reference:** We inserted explicit in-text citations to Figure 8 where its contents are discussed, ensuring production can generate the appropriate links.
- **Table cross-references (Tables 1 & 8):** We added clear in-text citations to Table 1 (Methods/Data Collection) and Table 8 (Results) so the tables are properly linked in production.

We thank the Reviewer and Academic Editor again for the helpful comments that significantly improved our work. We hope the revisions address all concerns satisfactorily.

Sincerely,
**[Corresponding Author's Name]** Parkpoom Chaisiriprasert, Apicha Deearom
**[Affiliation]** College of Digital Innovation Technology, Rangsit University
**[Email]** parkpoom.c@rsu.ac.th, apicha.d67@rsu.ac.th
