# Supplementary material for: A deep learning-based fusion framework for robust fine-grained classification of sea turtles in support of marine biodiversity
Source: PLoS One. 2026 Jun 9;21(6):e0344942. doi: 10.1371/journal.pone.0344942 (PMC13249215; doi:10.1371/journal.pone.0344942)
Supplement: Attachment — Submitted filename: [mje] Response to Reviewers.docx [file pone.0344942.s007.docx]

**Response to Reviewers**

**Manuscript Title:** A Deep Learning-Based Fusion Framework for Robust Fine-Grained Classification of Sea Turtles in Support of Marine Biodiversity
**Journal:** PLOS ONE
**Date:** [11/25/2025]

Dear Editor and Reviewers,

We would like to thank you for the time and effort spent reviewing our manuscript. We appreciate the insightful and constructive comments provided by Reviewer #1. These suggestions have significantly helped us improve the structure, logic, and clarity of our work.

We have carefully revised the manuscript in accordance with the reviewer’s comments. Below is a point-by-point response detailing the changes made.

### Response to Reviewer #1

**Comment 1:** Each section and its corresponding subsections should be hierarchically numbered to reflect the clear structure and logic of the paper.

**Response:** Thank you for this suggestion. We have restructured the entire manuscript to use hierarchical numbering. All primary sections (1. Introduction, 2. Materials and Methods, 3. Results and Discussion, 4. Conclusions) and their respective subsections (e.g., 2.1, 2.2.1, 2.2.2) are now clearly numbered to improve readability and logical flow.

**Comment 2:** The description of Figure 2 is incomplete, disorganized and not comprehensive. The description from line 174 to 183 on page 6 is incomplete. Obviously, the descriptions of the training and testing phases are missing.

**Response:** We have rewritten the description of the proposed framework to be more comprehensive. Specifically, we have expanded Section 2.2 Proposed Method to explicitly describe the three stages illustrated in Figure 2: (1) Preprocessing, (2) Fusion/Backbone, and (3) Training/Testing. Furthermore, in Section 2.2.6 (Training and Testing), we have added a detailed explanation of the operational flow shown in Figure 2, distinguishing between the "upper path" used for training (optimizing the fusion module and backbone) and the "lower path" used for inference/testing.

**Comment 3:** The sequence of the subsections under the section "Proposed method" is disordered. It is recommended to follow the image processing process described in Figure 2 and arrange the sub-section titles in the order from top to bottom and from left to right.

**Response:** We agree that the structure should mirror the pipeline. We have reorganized Section 2.2 to strictly follow the data flow presented in Figure 2:

- **2.2.1 Sobel Filtering** (Preprocessing)
- **2.2.2 Color Histogram Selection** (Preprocessing)
- **2.2.3 Tensor Encode** (Encoding)
- **2.2.4 Fusion Strategy** (LiteAFNet and AlphaBlendNet)
- **2.2.5 ResNet-50 and Modification** (Backbone)
- **2.2.6 Training and Testing** (Final Output)

**Comment 4:** The section "Parameter-Level Comparison of Fusion Modules" is in an inappropriate position in the paper. It belongs to the experimental analysis results and should not be placed under "proposed method".

**Response:** We have moved this analysis from the Methodology section to the Results and Discussion section. It is now located in Section 3.3.1 Parameter-Level Comparison of Fusion Modules. This placement allows us to discuss the parameter efficiency alongside the quantitative performance metrics (FLOPs and Latency).

**Comment 5:** In the Proposed methods section, the author states that LiteAFNet and AlphaBlend Net belong to the early stages of the entire network and should be trained simultaneously with Res-Net50. However, in Fig. 2, Train is marked after it. Please mark it before to avoid ambiguity.

**Response:** We have revised Figure 2 and the accompanying text in Section 2.2.6 to clearly indicate that the Fusion Strategy is part of the end-to-end training pipeline. The diagram now visually groups the fusion modules within the training block to show that they are optimized jointly with the backbone.

**Comment 6:** In Table 2, the increase value of the parameter seems to be incorrect. Please provide the correct value or explain the calculation method of Additional Params.

**Response:** We appreciate this careful check. In the revised manuscript, the parameter comparison table has been renumbered and is now Table 5 “Parameter comparison between conventional attention modules and the proposed fusion modules integrated with ResNet-50”.

We recomputed and corrected all parameter values using torchinfo on the implemented PyTorch models with a 4-channel input (RGB + Sobel) and histogram branch. The updated entries are:

- **ResNet-50 (Backbone):** 24.82663 M parameters
- **SE Block:** 24.82663 M (Additional Params: +0.00000 M)
- **CBAM:** 24.82673 M (Additional Params: +0.00010 M)
- **LiteAFNet (Ours):** 24.82665 M (Additional Params: +0.00002 M)
- **AlphaBlendNet (Ours):** 24.82664 M (Additional Params: +0.00001 M)

**Comment 7:** It is best to change the "Learning Rate Decay" in Table 4 to "Learning Rate Decay Strategy" to ensure the accuracy of the description.

**Response:** We have made this correction. In Table 3 (formerly Table 4), the parameter is now labeled "Learning Rate Decay Strategy," and the value is specified as "ReduceLROnPlateau."

**Comment 8:** The conclusion of the paper did not discuss the limitations of the research, practical applications of ecology, and future improvement directions.

**Response:** We agree that the conclusion should more explicitly address limitations, ecological applications, and future work. The Conclusions section (Section 4) has been substantially expanded and reorganized into three parts:

1. **Summary of contributions and main findings:**
   - We restate that integrating RGB, Sobel edge structure, and color histograms with adaptive fusion (LiteAFNet and AlphaBlendNet) significantly improves fine-grained sea turtle classification.
   - We summarize the key macro metrics, emphasizing that AlphaBlendNet achieved F1-score 0.86 and mAP 87.2%, with LiteAFNet providing an accuracy–efficiency trade-off.
2. **Practical ecological applications:**
   - We now explicitly discuss how the framework can support coastal and reef monitoring, UAV-based surveys, and fixed camera systems, helping to reduce manual annotation burdens and enabling high-frequency monitoring of sea turtle presence and abundance.
   - We explain how integrating automated classification with spatiotemporal metadata can assist in tracking habitat use, migration patterns, and ecological responses to environmental changes.
   - We highlight that LiteAFNet is particularly suited for edge deployment on low-power field devices, whereas AlphaBlendNet serves as a high-precision backend for large archival datasets.
3. **Limitations and future improvement directions:**
   We now explicitly list several limitations:
   - Dataset limitations: The current dataset is restricted to seven sea turtle species and may not fully capture global underwater variability (different water qualities, regions, and imaging conditions).
   - Domain generalization: We note that generalization to extreme turbidity or night-time imagery has not been fully tested and requires further validation.
   - Single-frame modeling: The present framework operates on single images and does not yet exploit temporal information from videos, which could help with occlusions and ambiguous views.
   - Hardware diversity: FLOPs and latency were measured on a single GPU platform; more comprehensive benchmarking across heterogeneous hardware (embedded GPUs, CPUs, edge accelerators) is needed.
   - Human-in-the-loop validation: While we used Grad-CAM for visual interpretability, we acknowledge that formal user studies with marine biologists are needed to rigorously evaluate trust and usability.

For future work, we now explicitly state that:

- We will broaden the taxonomic scope primarily by augmenting data with additional sea turtle subpopulations, and then extend to freshwater turtle species (Order Testudines) to test generalization across different aquatic ecosystems.
- We plan to integrate temporal modeling (e.g., transformer-based video architectures) to leverage motion continuity.
- We will explore semi-supervised domain adaptation to mitigate domain shifts during long-term monitoring.
- We aim to optimize the models for autonomous platforms such as underwater vehicles or smart buoys to enable large-scale, field-ready biodiversity monitoring.

We thank the Reviewer and Academic Editor again for the helpful comments that significantly improved our work. We hope the revisions address all concerns satisfactorily.

Sincerely,
**[Corresponding Author’s Name]** Parkpoom Chaisiriprasert, Apicha Deearom
**[Affiliation]** College of Digital Innovation Technology, Rangsit University
**[Email]** parkpoom.c@rsu.ac.th, apicha.d67@rsu.ac.th
