# Supplementary material for: A deep learning-based fusion framework for robust fine-grained classification of sea turtles in support of marine biodiversity
Source: PLoS One. 2026 Jun 9;21(6):e0344942. doi: 10.1371/journal.pone.0344942 (PMC13249215; doi:10.1371/journal.pone.0344942)
Supplement: Attachment — Submitted filename: Response to Reviewers.docx [file pone.0344942.s008.docx]

**Response to Reviewers**

**Manuscript Title:** A Deep Learning-Based Fusion Framework for Robust Fine-Grained Classification of Sea Turtles in Support of Marine Biodiversity
**Journal:** PLOS ONE
**Date:** [01/29/2026]

Dear Editor and Reviewers,

We would like to thank you for the time and effort spent reviewing our manuscript. We appreciate the insightful and constructive comments provided by Reviewer #2 and Reviewer #3. These suggestions have significantly helped us improve the structure, logic, and clarity of our work.

We have carefully revised the manuscript in accordance with the reviewer’s comments. Below is a point-by-point response detailing the changes made.

### Response to Reviewer #2

**Comment 1:** You mention that the image data were sourced from publicly available feature that debended in the study, are you mean (the properties in the dataset), if that, which feature you depend on your study, and if you reduce dimention of the feature, what is the method used to reduce the dimention of the feature, and why choose these. if not, so need to clarify the meaning of (the image data were sourced from publicly available feature)

**Response:** Thank you for this comment. The wording “publicly available feature” in the previous version was unclear and could be misinterpreted. The manuscript has been revised to clarify that the dataset was collected from publicly available repositories (Roboflow and seaturtle.org) and consists of raw images accompanied by annotation files containing the species label and bounding box coordinates for the annotated turtle. No pre-computed feature vectors were used from the data sources. Instead, feature representations were learned directly from the raw images by the proposed deep learning model; therefore, no feature dimensionality reduction (e.g., PCA) was performed.

**Change made:** The ambiguous statement was replaced with a concise description of the dataset contents (raw images + annotation files with species labels and bounding boxes) and an explicit statement that no dataset-provided feature vectors and no dimensionality reduction were used.

**Comment 2:** The paper mention to sobel filter two times as a subtitle, should be not.

**Response:** Thank you for the comment. The manuscript previously mentioned “Sobel” as a subsection title in more than one place, which could be redundant. To improve clarity, the subsection titles were revised to avoid repetition by renaming them to “Sobel Filtering” (Methods) and “Sobel Edge Result” (Results), while retaining the Sobel description within the text.

**Comment 3:** The study should clearer explanation about why use sobel not anothers.

**Response:** Thank you for the suggestion. A clearer justification for selecting Sobel was added in the Methods section. Sobel was chosen because it provides a lightweight, parameter-free, and stable gradient-based edge representation with minimal tuning, which is suitable for efficient downstream fusion and classification.

**Comment 4:** The sequence of the subsections under the section 'Proposed method' is disordered

**Response:** We agree that the previous ordering could be improved. The subsections under “Proposed method” were reorganized to follow the pipeline more logically:

- **2.2.1 Sobel Filtering** (Preprocessing)
- **2.2.2 Color Histogram Selection** (Preprocessing)
- **2.2.3 Tensor Encode** (Encoding)
- **2.2.4 Fusion Strategy** (LiteAFNet and AlphaBlendNet)
- **2.2.5 ResNet-50 and Modification** (Backbone)
- **2.2.6 Training and Testing**

**Comment 5:** The "Parameter-Level Comparison of Fusion Modules" section should not be placed under "proposed method".

**Response:** We have moved this analysis from the Methodology section to the Results and Discussion section. It is now located in Section 3.3.1 Parameter-Level Comparison of Fusion Modules. This placement allows us to discuss the parameter efficiency alongside the quantitative performance metrics (FLOPs and Latency).

**Comment 6:** The conclusion should be reformulated to be clearer, did not discuss the limitations of the research, practical applications of ecology, and should referring to the results obtained and on which the methods were compared to arrive at the best method.

**Response:** Thank you for the constructive suggestion. The Conclusions section has been reformulated to more clearly summarize the main findings and explicitly link the final claims to the quantitative results. The revised text now states that AlphaBlendNet achieved the best overall performance (macro F1-score 0.86, mAP 87.2%) compared with LiteAFNet (macro F1-score 0.80, mAP 83.4%) and Baseline Fusion (macro F1-score 0.72, mAP 75.9%), while also noting the substantially lower macro-averaged performance of lightweight backbones (MobileNetV3-Large and EfficientNet-B0) in this setting. In addition, practical ecological applications (photo-based surveys, archive screening, and consistent species-level reporting) have been added. Finally, the revised conclusion explicitly lists key limitations (public data sources/annotation quality, diversity of conditions, still-image evaluation, and lack of external field validation) and outlines concrete directions for future work (expanded data coverage, cross-site validation, domain adaptation, and temporal modeling).

**Change made:** Section 4 (Conclusions) was rewritten to include result-grounded comparisons, ecological applications, limitations, and future work.

### Response to Reviewer #3

**Comment 1:** The authors have made substantial efforts to enhance the manuscript based on the previous reviewers comments. However, I would suggest providing raw data and scripts attached as a supplement or shared through a sharing platform like git to ensure transparency and repeatability of the analysis. In addition, postpublication peer reviewers will require these raw data and raw scripts for independant verification of the claims.

**Response:** Thank you for this important suggestion regarding transparency and independent verification. In response, we have made the complete scripts used for preprocessing and training (including Baseline Fusion, LiteAFNet, and AlphaBlendNet), along with dependency specifications and usage instructions, publicly available through a Git repository: <https://github.com/parkpoom-c/LiteAFNet-and-AlphaBlendNet-.git >.

In addition, we have expanded the Supporting Information to improve accessibility of the materials required for verification. Specifically, we provide sample annotated images covering all seven sea turtle species (S3 Data), and we add the following Supporting Information item: S4 Code. Training and preprocessing scripts, which points to the public repository containing the raw scripts and instructions. We also updated the manuscript’s Data/Code Availability statement accordingly to clearly indicate where readers and post-publication reviewers can access these resources for independent assessment of our claims.

We thank the Reviewer and Academic Editor again for the helpful comments that significantly improved our work. We hope the revisions address all concerns satisfactorily.

Sincerely,
**[Corresponding Author’s Name]** Parkpoom Chaisiriprasert, Apicha Deearom
**[Affiliation]** College of Digital Innovation Technology, Rangsit University
**[Email]** parkpoom.c@rsu.ac.th, apicha.d@rsu.ac.th
