# Supplementary material for: A deep learning-based fusion framework for robust fine-grained classification of sea turtles in support of marine biodiversity
Source: PLoS One. 2026 Jun 9;21(6):e0344942. doi: 10.1371/journal.pone.0344942 (PMC13249215; doi:10.1371/journal.pone.0344942)
Supplement: Peer review history

# Peer review: Decision Letter 0

**DOI:** 10.1371/journal.pone.0344942.r001

**Authors:** Yaseen Ahmed Al-Mulla

2 Oct 2025

PONE-D-25-44025

A Deep Learning-Based Fusion Framework for Robust Fine-Grained Classification of Sea Turtles in Support of Marine Biodiversity

PLOS ONE

Dear Dr. Chaisiriprasert,

Thank you for submitting your manuscript to PLOS ONE. After careful consideration, we feel that it has merit but does not fully meet PLOS ONE’s publication criteria as it currently stands. Therefore, we invite you to submit a revised version of the manuscript that addresses the points raised during the review process.

Please submit your revised manuscript by Nov 16 2025 11:59PM. If you will need more time than this to complete your revisions, please reply to this message or contact the journal office at plosone@plos.org. When you're ready to submit your revision, log on to https://www.editorialmanager.com/pone/ and select the 'Submissions Needing Revision' folder to locate your manuscript file.

Please include the following items when submitting your revised manuscript:

- A rebuttal letter that responds to each point raised by the academic editor and reviewer(s). You should upload this letter as a separate file labeled 'Response to Reviewers'.
- A marked-up copy of your manuscript that highlights changes made to the original version. You should upload this as a separate file labeled 'Revised Manuscript with Track Changes'.
- An unmarked version of your revised paper without tracked changes. You should upload this as a separate file labeled 'Manuscript'.

-->If you would like to make changes to your financial disclosure, please include your updated statement in your cover letter. Guidelines for resubmitting your figure files are available below the reviewer comments at the end of this letter.

If applicable, we recommend that you deposit your laboratory protocols in protocols.io to enhance the reproducibility of your results. Protocols.io assigns your protocol its own identifier (DOI) so that it can be cited independently in the future. For instructions see: https://journals.plos.org/plosone/s/submission-guidelines#loc-laboratory-protocols. Additionally, PLOS ONE offers an option for publishing peer-reviewed Lab Protocol articles, which describe protocols hosted on protocols.io. Read more information on sharing protocols at https://plos.org/protocols?utm_medium=editorial-email&utm_source=authorletters&utm_campaign=protocols.

We look forward to receiving your revised manuscript.

Kind regards,

Yaseen Al-Mulla

Academic Editor

PLOS ONE

Journal Requirements:

When submitting your revision, we need you to address these additional requirements.

1. Please ensure that your manuscript meets PLOS ONE's style requirements, including those for file naming. The PLOS ONE style templates can be found at

https://journals.plos.org/plosone/s/file?id=wjVg/PLOSOne_formatting_sample_main_body.pdf and

https://journals.plos.org/plosone/s/file?id=ba62/PLOSOne_formatting_sample_title_authors_affiliations.pdf

2.  Please note that PLOS One has specific guidelines on code sharing for submissions in which author-generated code underpins the findings in the manuscript. In these cases, we expect all author-generated code to be made available without restrictions upon publication of the work. Please review our guidelines at https://journals.plos.org/plosone/s/materials-and-software-sharing#loc-sharing-code and ensure that your code is shared in a way that follows best practice and facilitates reproducibility and reuse.

3. Thank you for stating the following in your Competing Interests section: “No”

Please complete your Competing Interests on the online submission form to state any Competing Interests. If you have no competing interests, please state "The authors have declared that no competing interests exist.", as detailed online in our guide for authors at http://journals.plos.org/plosone/s/submit-now

This information should be included in your cover letter; we will change the online submission form on your behalf.

4. Please ensure that you refer to Figure 8 in your text as, if accepted, production will need this reference to link the reader to the figure.

5. We note you have included a table to which you do not refer in the text of your manuscript. Please ensure that you refer to Table 1 and 8 in your text; if accepted, production will need this reference to link the reader to the Table.

6. Please upload a copy of Supporting Information Figure/Table/etc. “Supporting information” which you refer to in your text on page 32 in PDF submission.

7. If the reviewer comments include a recommendation to cite specific previously published works, please review and evaluate these publications to determine whether they are relevant and should be cited. There is no requirement to cite these works unless the editor has indicated otherwise.

8. Please review your reference list to ensure that it is complete and correct. If you have cited papers that have been retracted, please include the rationale for doing so in the manuscript text, or remove these references and replace them with relevant current references. Any changes to the reference list should be mentioned in the rebuttal letter that accompanies your revised manuscript. If you need to cite a retracted article, indicate the article’s retracted status in the References list and also include a citation and full reference for the retraction notice.

[Note: HTML markup is below. Please do not edit.]

Reviewers' comments:

Reviewer's Responses to Questions

**Comments to the Author**

1. Is the manuscript technically sound, and do the data support the conclusions?

The manuscript must describe a technically sound piece of scientific research with data that supports the conclusions. Experiments must have been conducted rigorously, with appropriate controls, replication, and sample sizes. The conclusions must be drawn appropriately based on the data presented.

Reviewer #1: Yes

**********

2. Has the statistical analysis been performed appropriately and rigorously?

Reviewer #1: Yes

**********

3. Have the authors made all data underlying the findings in their manuscript fully available?

The PLOS Data policy requires authors to make all data underlying the findings described in their manuscript fully available without restriction, with rare exception (please refer to the Data Availability Statement in the manuscript PDF file). The data should be provided as part of the manuscript or its supporting information, or deposited to a public repository. For example, in addition to summary statistics, the data points behind means, medians and variance measures should be available. If there are restrictions on publicly sharing data—e.g. participant privacy or use of data from a third party—those must be specified.

Reviewer #1: Yes

**********

4. Is the manuscript presented in an intelligible fashion and written in standard English?

PLOS ONE does not copyedit accepted manuscripts, so the language in submitted articles must be clear, correct, and unambiguous. Any typographical or grammatical errors should be corrected at revision, so please note any specific errors here.

Reviewer #1: Yes

**********

5. Review Comments to the Author

Please use the space provided to explain your answers to the questions above. You may also include additional comments for the author, including concerns about dual publication, research ethics, or publication ethics. (Please upload your review as an attachment if it exceeds 20,000 characters)

Reviewer #1: This manuscript addresses the limitations of traditional manual visual classification, which is inefficient and highly sensitive to underwater imaging conditions. The authors propose a deep learning-based multimodal feature fusion framework that combines RGB color information and Sobel edge structural features for fine-grained classification of sea turtle species. The framework incorporates two custom-designed modules, LiteAFNet and AlphaBlendNet, and employs a modified ResNet-50 backbone for feature extraction. The core contribution lies in designing a robust classification system to overcome underwater imaging challenges. Experimental results demonstrate that, compared to the baseline approach, both fusion modules significantly improve classification performance.

However, there remain areas where the manuscript could be improved. I recommend minor revision before acceptance, with attention to the following points:

1. In the sections describing LiteAFNet and AlphaBlendNet, it would be beneficial to include parameter-level comparisons with other similar modules. For instance, since LiteAFNet is introduced as a lightweight attention-based fusion module, it could be compared with other attention mechanisms in terms of parameter count.

2. The dataset size is relatively small; the authors are encouraged to expand the dataset or validate the proposed framework on additional datasets to demonstrate its generalization capability.

3. In Table 3 of the Training section, there appears to be a typographical error. The correct training parameters should be provided.

4. Additional comparisons with other commonly used models would strengthen the experimental evaluation.

**********

6. PLOS authors have the option to publish the peer review history of their article (what does this mean?). If published, this will include your full peer review and any attached files.

If you choose “no”, your identity will remain anonymous but your review may still be made public.

**Do you want your identity to be public for this peer review?** For information about this choice, including consent withdrawal, please see our Privacy Policy.

Reviewer #1: No

**********

[NOTE: If reviewer comments were submitted as an attachment file, they will be attached to this email and accessible via the submission site. Please log into your account, locate the manuscript record, and check for the action link "View Attachments". If this link does not appear, there are no attachment files.]

While revising your submission, please upload your figure files to the Preflight Analysis and Conversion Engine (PACE) digital diagnostic tool, https://pacev2.apexcovantage.com/. PACE helps ensure that figures meet PLOS requirements. To use PACE, you must first register as a user. Registration is free. Then, login and navigate to the UPLOAD tab, where you will find detailed instructions on how to use the tool. If you encounter any issues or have any questions when using PACE, please email PLOS at figures@plos.org. Please note that Supporting Information files do not need this step.

# Author comment: Author response to Decision Letter 1

**DOI:** 10.1371/journal.pone.0344942.r002

3 Nov 2025

Response to Reviewers

Manuscript Title: A Deep Learning-Based Fusion Framework for Robust Fine-Grained Classification of Sea Turtles in Support of Marine Biodiversity

Journal: PLOS ONE

Date: [10/24/2025]

Dear Academic Editor and Reviewer,

We thank you for your thoughtful and constructive feedback. We have revised the manuscript accordingly and believe the changes substantially improve clarity, rigor, and reproducibility. Below we respond point-by-point; all edits are marked in the Revised Manuscript with Track Changes.

Reviewer #1

Overall assessment

“The manuscript proposes a deep learning-based multimodal fusion framework (RGB + Sobel) with two custom modules (LiteAFNet, AlphaBlendNet) and a modified ResNet-50; both modules improve performance. Minor revision recommended.”

Response: We appreciate the positive evaluation and address each item below.

1) Parameter-level comparisons for LiteAFNet and AlphaBlendNet

Comment: Include parameter-level comparisons with similar attention/fusion modules (e.g., parameter count).

Response (revised): Completed. We added a focused subsection and a summary table that reports the Total parameters of the end-to-end model and Additional parameters introduced by each module under the same ResNet-50 backbone, together with the integration stage of each module. Specifically, the baseline ResNet-50 totals 23.5M params; integrating SE and CBAM yields totals of 26.02M and 26.62M, with module-only additions of +0.12M and +0.18M, respectively. In contrast, our LiteAFNet and AlphaBlendNet keep the model totals at ≈23.52M, introducing only +0.00002M and +0.00003M parameters, respectively i.e., a negligible overhead while still enabling spatial/pixel-level adaptivity. Integration stages are clarified in the table (SE: channel; CBAM: channel+spatial; LiteAFNet: spatial; AlphaBlendNet: pixel-level).

Change in manuscript: Added Section “Parameter-Level Comparison of Fusion Modules” and Table 2: Parameter comparison between conventional attention modules and the proposed fusion modules integrated with ResNet-50 (Total Params, Additional Params, Integration Stage); minor cross-references added in Methods.

2) Dataset size and generalization

Comment: The dataset is relatively small; expand or validate on additional datasets to demonstrate generalizability.

Response: Addressed. We explicitly acknowledge the limited dataset size and strengthened the validation in two ways that are now described in the manuscript:

1. Extensive data augmentation to mitigate overfitting and improve reliability—random rotation, flipping, cropping, and color jittering—applied consistently during training.

2. Stratified 5-fold cross-validation during training and evaluation, ensuring a balanced representation of all seven species in every fold. This protocol lets each image contribute to both training and validation across iterations and yields a more reliable estimate of generalization.

Across folds, performance remained stable with only minor variations, indicating strong generalization. AlphaBlendNet achieved F1 = 0.86 ± 0.02 and mAP = 87.2% ± 1.5%, followed by LiteAFNet with F1 = 0.80 ± 0.03 and mAP = 83.4% ± 1.8%. The low standard deviations confirm that both proposed fusion architectures maintain consistent performance across data partitions despite the constrained dataset size.

Change in manuscript: Added/expanded text in Data Collection (augmentation and CV protocol) and Quantitative Performance Metrics (5-fold CV results with mean ± SD).

3) Typographical error in Training (Table 3)

Comment: There appears to be a typographical error. Please provide the correct training parameters.

Response: Corrected. We have updated the training configuration and renumbered the table due to new material added in the Methods. The Parameter Settings are now:

• Epochs: 100

• Batch size: 16

• Initial learning rate: 0.0001

• Learning-rate schedule: ReduceLROnPlateau

• Input resolution: 224 × 224 × 4 (RGB + Sobel)

All in-text citations that previously referred to the old training table were updated to the new numbering.

Change in manuscript: Replaced the training table with Table 4. Parameter Settings used for model training.

4) Additional comparisons with commonly used models

Comment: Please include more widely used models to strengthen the evaluation.

Response: Completed. We integrated two widely adopted lightweight classifiers—MobileNetV3-Large and EfficientNet-B0—and trained/evaluated them under the same experimental protocol as our methods (identical training settings in Table 4 and the same input-fusion pipeline used in the main experiments). We report per-class and macro metrics and analyze accuracy–efficiency trade-offs.

• New per-class results.

o Table 9 (MobileNetV3-Large): per-class Precision/Recall/F1/mAP; macro F1 = 0.48 and mAP = 45.6%.

o Table 10 (EfficientNet-B0): per-class Precision/Recall/F1/mAP; macro F1 = 0.56 and mAP = 53.3%—higher than MobileNetV3-L across most species, but still below our learnable fusion modules.

o Figure 9: per-class mAP (%) curves for the seven species now compare Baseline Fusion, LiteAFNet, AlphaBlendNet, MobileNetV3-L, and EfficientNet-B0, showing the consistent advantage of LiteAFNet/AlphaBlendNet over all baselines.

• Efficiency analysis.

o Table 11 (FLOPs & latency): MobileNetV3-L = 0.47 GFLOPs / 20.55 ms, EfficientNet-B0 = 0.39 GFLOPs / 22.10 ms (single-image inference).

o Table 12 (per-class latency): per-species inference times reported for all models; MobileNetV3-L and EfficientNet-B0 remain in the ~20–22 ms range across classes.

• Macro-averaged summary.

o Table 13 now includes both new models alongside the existing methods:

• MobileNetV3-L: Precision 0.52, Recall 0.45, F1 = 0.48, mAP = 45.6%.

• EfficientNet-B0: Precision 0.58, Recall 0.54, F1 = 0.56, mAP = 53.3%.

• Both trail our LiteAFNet (F1 = 0.80, mAP = 83.4%) and AlphaBlendNet (F1 = 0.86, mAP = 87.2%), confirming that learnable fusion outperforms simple concatenation-based baselines under realistic underwater conditions.

Change in manuscript: Added Table 9 and Table 10 (per-class metrics); updated Figure 9 (per-class mAP curves), Table 11 (FLOPs & latency), Table 12 (per-class latency), and Table 13 (macro-averaged metrics). Corresponding narrative updates were made in Results and Discussion to incorporate MobileNetV3-Large and EfficientNet-B0.

Journal Requirements

• Reference list audit: We reviewed all references, added missing DOIs, corrected metadata, and replaced unverifiable entries with current, citable sources. The revised bibliography reflects these updates.

• Figure 8 cross-reference: We inserted explicit in-text citations to Figure 8 where its contents are discussed, ensuring production can generate the appropriate links.

• Table cross-references (Tables 1 & 8): We added clear in-text citations to Table 1 (Methods/Data Collection) and Table 8 (Results) so the tables are properly linked in production.

We thank the Reviewer and Academic Editor again for the helpful comments that significantly improved our work. We hope the revisions address all concerns satisfactorily.

Sincerely,

[Corresponding Author’s Name] Parkpoom Chaisiriprasert, Apicha Deearom

[Affiliation] College of Digital Innovation Technology, Rangsit University

[Email] parkpoom.c@rsu.ac.th, apicha.d67@rsu.ac.th

Attachment Submitted filename: Response to Reviewers.pdf

# Peer review: Decision Letter 1

**DOI:** 10.1371/journal.pone.0344942.r003

**Authors:** Yaseen Ahmed Al-Mulla

15 Nov 2025

PONE-D-25-44025R1

A Deep Learning-Based Fusion Framework for Robust Fine-Grained Classification of Sea Turtles in Support of Marine Biodiversity

PLOS ONE

Dear Dr. Chaisiriprasert,

Thank you for submitting your manuscript to PLOS ONE. After careful consideration, we feel that it has merit but does not fully meet PLOS ONE’s publication criteria as it currently stands. Therefore, we invite you to submit a revised version of the manuscript that addresses the points raised during the review process.

Please submit your revised manuscript by Dec 30 2025 11:59PM. If you will need more time than this to complete your revisions, please reply to this message or contact the journal office at plosone@plos.org. When you're ready to submit your revision, log on to https://www.editorialmanager.com/pone/ and select the 'Submissions Needing Revision' folder to locate your manuscript file.

Please include the following items when submitting your revised manuscript:

- A rebuttal letter that responds to each point raised by the academic editor and reviewer(s). You should upload this letter as a separate file labeled 'Response to Reviewers'.
- A marked-up copy of your manuscript that highlights changes made to the original version. You should upload this as a separate file labeled 'Revised Manuscript with Track Changes'.
- An unmarked version of your revised paper without tracked changes. You should upload this as a separate file labeled 'Manuscript'.

If you would like to make changes to your financial disclosure, please include your updated statement in your cover letter. Guidelines for resubmitting your figure files are available below the reviewer comments at the end of this letter.

If applicable, we recommend that you deposit your laboratory protocols in protocols.io to enhance the reproducibility of your results. Protocols.io assigns your protocol its own identifier (DOI) so that it can be cited independently in the future. For instructions see: https://journals.plos.org/plosone/s/submission-guidelines#loc-laboratory-protocols. Additionally, PLOS ONE offers an option for publishing peer-reviewed Lab Protocol articles, which describe protocols hosted on protocols.io. Read more information on sharing protocols at https://plos.org/protocols?utm_medium=editorial-email&utm_source=authorletters&utm_campaign=protocols.

We look forward to receiving your revised manuscript.

Kind regards,

Yaseen Al-Mulla

Academic Editor

PLOS ONE

Journal Requirements:

1. If the reviewer comments include a recommendation to cite specific previously published works, please review and evaluate these publications to determine whether they are relevant and should be cited. There is no requirement to cite these works unless the editor has indicated otherwise.

[Note: HTML markup is below. Please do not edit.]

Reviewer's Responses to Questions

**Comments to the Author**

1. If the authors have adequately addressed your comments raised in a previous round of review and you feel that this manuscript is now acceptable for publication, you may indicate that here to bypass the “Comments to the Author” section, enter your conflict of interest statement in the “Confidential to Editor” section, and submit your "Accept" recommendation.

Reviewer #1: (No Response)

**********

2. Is the manuscript technically sound, and do the data support the conclusions?

The manuscript must describe a technically sound piece of scientific research with data that supports the conclusions. Experiments must have been conducted rigorously, with appropriate controls, replication, and sample sizes. The conclusions must be drawn appropriately based on the data presented.

Reviewer #1: Partly

**********

3. Has the statistical analysis been performed appropriately and rigorously?

Reviewer #1: I Don't Know

**********

4. Have the authors made all data underlying the findings in their manuscript fully available?

The PLOS Data policy requires authors to make all data underlying the findings described in their manuscript fully available without restriction, with rare exception (please refer to the Data Availability Statement in the manuscript PDF file). The data should be provided as part of the manuscript or its supporting information, or deposited to a public repository. For example, in addition to summary statistics, the data points behind means, medians and variance measures should be available. If there are restrictions on publicly sharing data—e.g. participant privacy or use of data from a third party—those must be specified.

Reviewer #1: Yes

**********

5. Is the manuscript presented in an intelligible fashion and written in standard English?

PLOS ONE does not copyedit accepted manuscripts, so the language in submitted articles must be clear, correct, and unambiguous. Any typographical or grammatical errors should be corrected at revision, so please note any specific errors here.

Reviewer #1: Yes

**********

6. Review Comments to the Author

Please use the space provided to explain your answers to the questions above. You may also include additional comments for the author, including concerns about dual publication, research ethics, or publication ethics. (Please upload your review as an attachment if it exceeds 20,000 characters)

Reviewer #1: There are currently numerous issues with the writing of the thesis, and the organization of the thesis's logic is chaotic. It is recommended that the paper be revised to ensure that its writing quality meets the research article standards before it is submitted. The specific and more serious problems are as follows.

1. Each section and its corresponding subsections should be hierarchically numbered to reflect the clear structure and logic of the paper.

2. The description of Figure 2 is incomplete, disorganized and not comprehensive. The description from line 174 to 183 on page 6 is incomplete. Obviously, the descriptions of the training and testing phases are missing.

3. The sequence of the subsections under the section "Proposed method" is disordered. It is recommended to follow the image processing process described in Figure 2 and arrange the sub-section titles in the order from top to bottom and from left to right, such as: xx.1 Sobel Filtering, xx.2 Color Histogram Selection, xx.3 Tensor Encode, xx.4 Fusion, xx.4.1 LiteAFNet, xx.4.2 AlphaBlentNet, ……..

4. The section "Parameter-Level Comparison of Fusion Modules" is in an inappropriate position in the paper. It belongs to the experimental analysis results and should not be placed under "proposed method".

5.In the Proposed methods section, the author states that LiteAFNet and AlphaBlend Net belong to the early stages of the entire network and should be trained simultaneously with Res-Net50. However, in Fig. 2, Train is marked after it. Please mark it before to avoid ambiguity.

6.In Table 2, the increase value of the parameter seems to be incorrect. Please provide the correct value or explain the calculation method of Additional Params.

7.It is best to change the "Learning Rate Decay" in Table 4 to "Learning Rate Decay Strategy" to ensure the accuracy of the description.

8. The conclusion of the paper did not discuss the limitations of the research, practical applications of ecology, and future improvement directions.

**********

7. PLOS authors have the option to publish the peer review history of their article (what does this mean?). If published, this will include your full peer review and any attached files.

If you choose “no”, your identity will remain anonymous but your review may still be made public.

**Do you want your identity to be public for this peer review?** For information about this choice, including consent withdrawal, please see our Privacy Policy.

Reviewer #1: No

**********

[NOTE: If reviewer comments were submitted as an attachment file, they will be attached to this email and accessible via the submission site. Please log into your account, locate the manuscript record, and check for the action link "View Attachments". If this link does not appear, there are no attachment files.]

To ensure your figures meet our technical requirements, please review our figure guidelines: https://journals.plos.org/plosone/s/figures

You may also use PLOS’s free figure tool, NAAS, to help you prepare publication quality figures: https://journals.plos.org/plosone/s/figures#loc-tools-for-figure-preparation

NAAS will assess whether your figures meet our technical requirements by comparing each figure against our figure specifications.

# Author comment: Author response to Decision Letter 2

**DOI:** 10.1371/journal.pone.0344942.r004

26 Nov 2025

Response to Reviewers

Manuscript Title: A Deep Learning-Based Fusion Framework for Robust Fine-Grained Classification of Sea Turtles in Support of Marine Biodiversity

Journal: PLOS ONE

Date: [11/25/2025]

Dear Editor and Reviewers,

We would like to thank you for the time and effort spent reviewing our manuscript. We appreciate the insightful and constructive comments provided by Reviewer #1. These suggestions have significantly helped us improve the structure, logic, and clarity of our work.

We have carefully revised the manuscript in accordance with the reviewer’s comments. Below is a point-by-point response detailing the changes made.

Response to Reviewer #1

Comment 1: Each section and its corresponding subsections should be hierarchically numbered to reflect the clear structure and logic of the paper.

Response: Thank you for this suggestion. We have restructured the entire manuscript to use hierarchical numbering. All primary sections (1. Introduction, 2. Materials and Methods, 3. Results and Discussion, 4. Conclusions) and their respective subsections (e.g., 2.1, 2.2.1, 2.2.2) are now clearly numbered to improve readability and logical flow.

Comment 2: The description of Figure 2 is incomplete, disorganized and not comprehensive. The description from line 174 to 183 on page 6 is incomplete. Obviously, the descriptions of the training and testing phases are missing.

Response: We have rewritten the description of the proposed framework to be more comprehensive. Specifically, we have expanded Section 2.2 Proposed Method to explicitly describe the three stages illustrated in Figure 2: (1) Preprocessing, (2) Fusion/Backbone, and (3) Training/Testing. Furthermore, in Section 2.2.6 (Training and Testing), we have added a detailed explanation of the operational flow shown in Figure 2, distinguishing between the "upper path" used for training (optimizing the fusion module and backbone) and the "lower path" used for inference/testing.

Comment 3: The sequence of the subsections under the section "Proposed method" is disordered. It is recommended to follow the image processing process described in Figure 2 and arrange the sub-section titles in the order from top to bottom and from left to right.

Response: We agree that the structure should mirror the pipeline. We have reorganized Section 2.2 to strictly follow the data flow presented in Figure 2:

• 2.2.1 Sobel Filtering (Preprocessing)

• 2.2.2 Color Histogram Selection (Preprocessing)

• 2.2.3 Tensor Encode (Encoding)

• 2.2.4 Fusion Strategy (LiteAFNet and AlphaBlendNet)

• 2.2.5 ResNet-50 and Modification (Backbone)

• 2.2.6 Training and Testing (Final Output)

Comment 4: The section "Parameter-Level Comparison of Fusion Modules" is in an inappropriate position in the paper. It belongs to the experimental analysis results and should not be placed under "proposed method".

Response: We have moved this analysis from the Methodology section to the Results and Discussion section. It is now located in Section 3.3.1 Parameter-Level Comparison of Fusion Modules. This placement allows us to discuss the parameter efficiency alongside the quantitative performance metrics (FLOPs and Latency).

Comment 5: In the Proposed methods section, the author states that LiteAFNet and AlphaBlend Net belong to the early stages of the entire network and should be trained simultaneously with Res-Net50. However, in Fig. 2, Train is marked after it. Please mark it before to avoid ambiguity.

Response: We have revised Figure 2 and the accompanying text in Section 2.2.6 to clearly indicate that the Fusion Strategy is part of the end-to-end training pipeline. The diagram now visually groups the fusion modules within the training block to show that they are optimized jointly with the backbone.

Comment 6: In Table 2, the increase value of the parameter seems to be incorrect. Please provide the correct value or explain the calculation method of Additional Params.

Response: We appreciate this careful check. In the revised manuscript, the parameter comparison table has been renumbered and is now Table 5 “Parameter comparison between conventional attention modules and the proposed fusion modules integrated with ResNet-50”.

We recomputed and corrected all parameter values using torchinfo on the implemented PyTorch models with a 4-channel input (RGB + Sobel) and histogram branch. The updated entries are:

• ResNet-50 (Backbone): 24.82663 M parameters

• SE Block: 24.82663 M (Additional Params: +0.00000 M)

• CBAM: 24.82673 M (Additional Params: +0.00010 M)

• LiteAFNet (Ours): 24.82665 M (Additional Params: +0.00002 M)

• AlphaBlendNet (Ours): 24.82664 M (Additional Params: +0.00001 M)

Comment 7: It is best to change the "Learning Rate Decay" in Table 4 to "Learning Rate Decay Strategy" to ensure the accuracy of the description.

Response: We have made this correction. In Table 3 (formerly Table 4), the parameter is now labeled "Learning Rate Decay Strategy," and the value is specified as "ReduceLROnPlateau."

Comment 8: The conclusion of the paper did not discuss the limitations of the research, practical applications of ecology, and future improvement directions.

Response: We agree that the conclusion should more explicitly address limitations, ecological applications, and future work. The Conclusions section (Section 4) has been substantially expanded and reorganized into three parts:

1. Summary of contributions and main findings:

• We restate that integrating RGB, Sobel edge structure, and color histograms with adaptive fusion (LiteAFNet and AlphaBlendNet) significantly improves fine-grained sea turtle classification.

• We summarize the key macro metrics, emphasizing that AlphaBlendNet achieved F1-score 0.86 and mAP 87.2%, with LiteAFNet providing an accuracy–efficiency trade-off.

2. Practical ecological applications:

• We now explicitly discuss how the framework can support coastal and reef monitoring, UAV-based surveys, and fixed camera systems, helping to reduce manual annotation burdens and enabling high-frequency monitoring of sea turtle presence and abundance.

• We explain how integrating automated classification with spatiotemporal metadata can assist in tracking habitat use, migration patterns, and ecological responses to environmental changes.

• We highlight that LiteAFNet is particularly suited for edge deployment on low-power field devices, whereas AlphaBlendNet serves as a high-precision backend for large archival datasets.

3. Limitations and future improvement directions:

We now explicitly list several limitations:

• Dataset limitations: The current dataset is restricted to seven sea turtle species and may not fully capture global underwater variability (different water qualities, regions, and imaging conditions).

• Domain generalization: We note that generalization to extreme turbidity or night-time imagery has not been fully tested and requires further validation.

• Single-frame modeling: The present framework operates on single images and does not yet exploit temporal information from videos, which could help with occlusions and ambiguous views.

• Hardware diversity: FLOPs and latency were measured on a single GPU platform; more comprehensive benchmarking across heterogeneous hardware (embedded GPUs, CPUs, edge accelerators) is needed.

• Human-in-the-loop validation: While we used Grad-CAM for visual interpretability, we acknowledge that formal user studies with marine biologists are needed to rigorously evaluate trust and usability.

For future work, we now explicitly state that:

• We will broaden the taxonomic scope primarily by augmenting data with additional sea turtle subpopulations, and then extend to freshwater turtle species (Order Testudines) to test generalization across different aquatic ecosystems.

• We plan to integrate temporal modeling (e.g., transformer-based video architectures) to leverage motion continuity.

• We will explore semi-supervised domain adaptation to mitigate domain shifts during long-term monitoring.

• We aim to optimize the models for autonomous platforms such as underwater vehicles or smart buoys to enable large-scale, field-ready biodiversity monitoring.

We thank the Reviewer and Academic Editor again for the helpful comments that significantly improved our work. We hope the revisions address all concerns satisfactorily.

Sincerely,

[Corresponding Author’s Name] Parkpoom Chaisiriprasert, Apicha Deearom

[Affiliation] College of Digital Innovation Technology, Rangsit University

[Email] parkpoom.c@rsu.ac.th, apicha.d67@rsu.ac.th

Attachment Submitted filename: [mje] Response to Reviewers.docx

# Peer review: Decision Letter 2

**DOI:** 10.1371/journal.pone.0344942.r005

**Authors:** Yaseen Ahmed Al-Mulla

26 Dec 2025

PONE-D-25-44025R2

A Deep Learning-Based Fusion Framework for Robust Fine-Grained Classification of Sea Turtles in Support of Marine Biodiversity

PLOS One

Dear Dr. Chaisiriprasert,

Thank you for submitting your manuscript to PLOS ONE. After careful consideration, we feel that it has merit but does not fully meet PLOS ONE’s publication criteria as it currently stands. Therefore, we invite you to submit a revised version of the manuscript that addresses the points raised during the review process.

Please submit your revised manuscript by Feb 09 2026 11:59PM. If you will need more time than this to complete your revisions, please reply to this message or contact the journal office at plosone@plos.org. When you're ready to submit your revision, log on to https://www.editorialmanager.com/pone/ and select the 'Submissions Needing Revision' folder to locate your manuscript file.

Please include the following items when submitting your revised manuscript:

- A letter that responds to each point raised by the academic editor and reviewer(s). You should upload this letter as a separate file labeled 'Response to Reviewers'.
- A marked-up copy of your manuscript that highlights changes made to the original version. You should upload this as a separate file labeled 'Revised Manuscript with Track Changes'.
- An unmarked version of your revised paper without tracked changes. You should upload this as a separate file labeled 'Manuscript'.

-->If you would like to make changes to your financial disclosure, please include your updated statement in your cover letter. Guidelines for resubmitting your figure files are available below the reviewer comments at the end of this letter.

If applicable, we recommend that you deposit your laboratory protocols in protocols.io to enhance the reproducibility of your results. Protocols.io assigns your protocol its own identifier (DOI) so that it can be cited independently in the future. For instructions see: https://journals.plos.org/plosone/s/submission-guidelines#loc-laboratory-protocols. Additionally, PLOS ONE offers an option for publishing peer-reviewed Lab Protocol articles, which describe protocols hosted on protocols.io. Read more information on sharing protocols at https://plos.org/protocols?utm_medium=editorial-email&utm_source=authorletters&utm_campaign=protocols.

We look forward to receiving your revised manuscript.

Kind regards,

Yaseen Al-Mulla

Academic Editor

PLOS One

Journal Requirements:

1. If the reviewer comments include a recommendation to cite specific previously published works, please review and evaluate these publications to determine whether they are relevant and should be cited. There is no requirement to cite these works unless the editor has indicated otherwise.

2. Please review your reference list to ensure that it is complete and correct. If you have cited papers that have been retracted, please include the rationale for doing so in the manuscript text, or remove these references and replace them with relevant current references. Any changes to the reference list should be mentioned in the rebuttal letter that accompanies your revised manuscript. If you need to cite a retracted article, indicate the article’s retracted status in the References list and also include a citation and full reference for the retraction notice.

[Note: HTML markup is below. Please do not edit.]

Reviewers' comments:

Reviewer's Responses to Questions

**Comments to the Author**

1. If the authors have adequately addressed your comments raised in a previous round of review and you feel that this manuscript is now acceptable for publication, you may indicate that here to bypass the “Comments to the Author” section, enter your conflict of interest statement in the “Confidential to Editor” section, and submit your "Accept" recommendation.

Reviewer #2: (No Response)

Reviewer #3: All comments have been addressed

**********

2. Is the manuscript technically sound, and do the data support the conclusions?

The manuscript must describe a technically sound piece of scientific research with data that supports the conclusions. Experiments must have been conducted rigorously, with appropriate controls, replication, and sample sizes. The conclusions must be drawn appropriately based on the data presented.

Reviewer #2: Yes

Reviewer #3: Yes

**********

3. Has the statistical analysis been performed appropriately and rigorously?

Reviewer #2: Yes

Reviewer #3: Yes

**********

4. Have the authors made all data underlying the findings in their manuscript fully available?

The PLOS Data policy requires authors to make all data underlying the findings described in their manuscript fully available without restriction, with rare exception (please refer to the Data Availability Statement in the manuscript PDF file). The data should be provided as part of the manuscript or its supporting information, or deposited to a public repository. For example, in addition to summary statistics, the data points behind means, medians and variance measures should be available. If there are restrictions on publicly sharing data—e.g. participant privacy or use of data from a third party—those must be specified.

Reviewer #2: Yes

Reviewer #3: No

**********

5. Is the manuscript presented in an intelligible fashion and written in standard English?

PLOS ONE does not copyedit accepted manuscripts, so the language in submitted articles must be clear, correct, and unambiguous. Any typographical or grammatical errors should be corrected at revision, so please note any specific errors here.

Reviewer #2: Yes

Reviewer #3: Yes

**********

6. Review Comments to the Author

Please use the space provided to explain your answers to the questions above. You may also include additional comments for the author, including concerns about dual publication, research ethics, or publication ethics. (Please upload your review as an attachment if it exceeds 20,000 characters)

Reviewer #2: The manuscript technically sounds good and has statistical analysis appropriately and rigorously presented in an intelligible fashion, but you need to adjust/modify the paper following the article journal template. See the published PDF paper.

1- you mention that the image data were sourced from publicly available feature that debended in the study, are you mean (the properties in the dataset), if that, which feature you depend on your study, and if you reduce dimention of the feature, what is the method used to reduce the dimention of the feature, and why choose these. if not, so need to clarify the meaning of (the image data were sourced from publicly available feature )

2- the paper mention to sobel filter two times as a subtitle, should be not.

3- the study should clearer explanation about why use sobel not anothers.

4-The sequence of the subsections under the section 'Proposed method' is disordered

5- The "Parameter-Level Comparison of Fusion Modules" section should not be placed under "proposed method".

6- The conclusion should be reformulated to be clearer, did not discuss the limitations of the research, practical applications of ecology, and should referring to the results obtained and on which the methods were compared to arrive at the best method.

Reviewer #3: The authors have made substantial efforts to enhance the manuscript based on the previous reviewers comments. However, I would suggest providing raw data and scripts attached as a supplement or shared through a sharing platform like git to ensure transparency and repeatability of the analysis. In addition, postpublication peer reviewers will require these raw data and raw scripts for independant verification of the claims.

**********

7. PLOS authors have the option to publish the peer review history of their article (what does this mean?). If published, this will include your full peer review and any attached files.

If you choose “no”, your identity will remain anonymous but your review may still be made public.

**Do you want your identity to be public for this peer review?** For information about this choice, including consent withdrawal, please see our Privacy Policy.

Reviewer #2: **Yes:** Nada Al-okbi

Reviewer #3: **Yes:** Thiru Somasundaram

**********

[NOTE: If reviewer comments were submitted as an attachment file, they will be attached to this email and accessible via the submission site. Please log into your account, locate the manuscript record, and check for the action link "View Attachments". If this link does not appear, there are no attachment files.]

To ensure your figures meet our technical requirements, please review our figure guidelines: https://journals.plos.org/plosone/s/figures

You may also use PLOS’s free figure tool, NAAS, to help you prepare publication quality figures: https://journals.plos.org/plosone/s/figures#loc-tools-for-figure-preparation.

NAAS will assess whether your figures meet our technical requirements by comparing each figure against our figure specifications.

# Author comment: Author response to Decision Letter 3

**DOI:** 10.1371/journal.pone.0344942.r006

30 Jan 2026

We have carefully revised the manuscript by thoroughly addressing all the reviewers’ comments and suggestions, which we believe have significantly improved the quality and clarity of the manuscript. A detailed, point-by-point response to the reviewers is provided in the response document, and all changes have been clearly indicated in the revised manuscript.

Attachment Submitted filename: Response to Reviewers.docx

# Peer review: Decision Letter 3

**DOI:** 10.1371/journal.pone.0344942.r007

**Authors:** Yaseen Ahmed Al-Mulla

27 Feb 2026

A Deep Learning-Based Fusion Framework for Robust Fine-Grained Classification of Sea Turtles in Support of Marine Biodiversity

PONE-D-25-44025R3

Dear Dr. Chaisiriprasert,

We’re pleased to inform you that your manuscript has been judged scientifically suitable for publication and will be formally accepted for publication once it meets all outstanding technical requirements.

Within one week, you’ll receive an e-mail detailing the required amendments. When these have been addressed, you’ll receive a formal acceptance letter and your manuscript will be scheduled for publication.

An invoice will be generated when your article is formally accepted. Please note, if your institution has a publishing partnership with PLOS and your article meets the relevant criteria, all or part of your publication costs will be covered. Please make sure your user information is up-to-date by logging into Editorial Manager at Editorial Manager® and clicking the ‘Update My Information' link at the top of the page. For questions related to billing, please contact billing support.

If your institution or institutions have a press office, please notify them about your upcoming paper to help maximize its impact. If they’ll be preparing press materials, please inform our press team as soon as possible -- no later than 48 hours after receiving the formal acceptance. Your manuscript will remain under strict press embargo until 2 pm Eastern Time on the date of publication. For more information, please contact onepress@plos.org.

Kind regards,

Yaseen Ahmed Al-Mulla

Academic Editor

PLOS One

Additional Editor Comments (optional):

Reviewers' comments:

Reviewer's Responses to Questions

**Comments to the Author**

1. If the authors have adequately addressed your comments raised in a previous round of review and you feel that this manuscript is now acceptable for publication, you may indicate that here to bypass the “Comments to the Author” section, enter your conflict of interest statement in the “Confidential to Editor” section, and submit your "Accept" recommendation.

Reviewer #2: All comments have been addressed

**********

2. Is the manuscript technically sound, and do the data support the conclusions?

The manuscript must describe a technically sound piece of scientific research with data that supports the conclusions. Experiments must have been conducted rigorously, with appropriate controls, replication, and sample sizes. The conclusions must be drawn appropriately based on the data presented.

Reviewer #2: Yes

**********

3. Has the statistical analysis been performed appropriately and rigorously?

Reviewer #2: Yes

**********

4. Have the authors made all data underlying the findings in their manuscript fully available?

The PLOS Data policy requires authors to make all data underlying the findings described in their manuscript fully available without restriction, with rare exception (please refer to the Data Availability Statement in the manuscript PDF file). The data should be provided as part of the manuscript or its supporting information, or deposited to a public repository. For example, in addition to summary statistics, the data points behind means, medians and variance measures should be available. If there are restrictions on publicly sharing data—e.g. participant privacy or use of data from a third party—those must be specified.

Reviewer #2: Yes

**********

5. Is the manuscript presented in an intelligible fashion and written in standard English?

PLOS ONE does not copyedit accepted manuscripts, so the language in submitted articles must be clear, correct, and unambiguous. Any typographical or grammatical errors should be corrected at revision, so please note any specific errors here.

Reviewer #2: Yes

**********

6. Review Comments to the Author

Please use the space provided to explain your answers to the questions above. You may also include additional comments for the author, including concerns about dual publication, research ethics, or publication ethics. (Please upload your review as an attachment if it exceeds 20,000 characters)

Reviewer #2: (No Response)

**********

7. PLOS authors have the option to publish the peer review history of their article (what does this mean?). If published, this will include your full peer review and any attached files.

If you choose “no”, your identity will remain anonymous but your review may still be made public.

**Do you want your identity to be public for this peer review?** For information about this choice, including consent withdrawal, please see our Privacy Policy.

Reviewer #2: No

**********

# Editor report: Acceptance letter

**DOI:** 10.1371/journal.pone.0344942.r008

**Authors:** Yaseen Ahmed Al-Mulla

PONE-D-25-44025R3

PLOS One

Dear Dr. Chaisiriprasert,

I'm pleased to inform you that your manuscript has been deemed suitable for publication in PLOS One. Congratulations! Your manuscript is now being handed over to our production team.

At this stage, our production department will prepare your paper for publication. This includes ensuring the following:

* All references, tables, and figures are properly cited

* All relevant supporting information is included in the manuscript submission,

* There are no issues that prevent the paper from being properly typeset

You will receive further instructions from the production team, including instructions on how to review your proof when it is ready. Please keep in mind that we are working through a large volume of accepted articles, so please give us a few days to review your paper and let you know the next and final steps.

Lastly, if your institution or institutions have a press office, please let them know about your upcoming paper now to help maximize its impact. If they'll be preparing press materials, please inform our press team within the next 48 hours. Your manuscript will remain under strict press embargo until 2 pm Eastern Time on the date of publication. For more information, please contact onepress@plos.org.

You will receive an invoice from PLOS for your publication fee after your manuscript has reached the completed accept phase. If you receive an email requesting payment before acceptance or for any other service, this may be a phishing scheme. Learn how to identify phishing emails and protect your accounts at https://explore.plos.org/phishing.

If we can help with anything else, please email us at customercare@plos.org.

Thank you for submitting your work to PLOS ONE and supporting open access.

Kind regards,

PLOS ONE Editorial Office Staff

on behalf of

Dr. Yaseen Ahmed Al-Mulla

Academic Editor

PLOS One
